# Integrative transcriptome analysis revealed the pathogenic molecular basis of *Rhizoctonia solani* AG-3 TB at three progressive stages of infection

**DOI:** 10.3389/fmicb.2022.1001327

**Published:** 2022-10-11

**Authors:** Xinchun Li, Mengnan An, Chuantao Xu, Lianqiang Jiang, Fangfang Yan, Yang Yang, Chong Zhang, Yuanhua Wu

**Affiliations:** ^1^Liaoning Key Laboratory of Plant Pathology, College of Plant Protection, Shenyang Agricultural University, Shenyang, China; ^2^Luzhou Branch of Sichuan Province Tobacco Company, Luzhou, China; ^3^Liangshan Branch of Sichuan Province Tobacco Company, Xichang, China; ^4^Panzhihua Branch of Sichuan Province Tobacco Company, Panzhihua, China; ^5^Yibin Branch of Sichuan Province Tobacco Company, Yibin, China

**Keywords:** *Rhizoctonia solani* AG-3 TB, pathogenic molecular mechanism, secondary metabolites, carbohydrate-active enzymes, cell wall degrading enzymes, effector

## Abstract

*Rhizoctonia solani* has a broad host range and results in significant losses in agricultural production. Here, an integrated transcriptomic analysis was performed to reveal the critical genes responsible for the pathogenesis of *R. solani* AG-3 TB on *Nicotiana tabacum* at different infection stages. The results showed that various differential expressed genes (DEGs) were enriched in fatty acid metabolism, amino sugar, carbon metabolism, and cellular carbohydrate biosynthetic process at the early (6–12 hpi), middle (24–36 hpi), and late stage (48–72 hpi) of infection. Specifically, several critical genes such as shikimate kinase that were involved in the biosynthesis of an important fungal toxin, phenylacetic acid (PAA) showed markedly increase at 24 hpi. Additionally, the genes expression levels of carbohydrate-active enzymes (CAZymes) and cell wall degrading enzymes (CWDEs) were significantly increased at the late infection stage. Furthermore, we identified 807 potential secreted proteins and 78 small cysteine-rich proteins, which may function as fungal effectors and involved in the pathogenicity. These results provide valuable insights into critical and potential genes as well as the pathways involved in the pathogenesis of *R. solani* AG-3 TB.

## Introduction

*Rhizoctonia solani* kühn (teleomorph: *Thanatephorus cucumeris*) belongs to the soil-borne basidiomycete and ubiquitously causes diseases on roots, stem and leaves of plant (Ogoshi, 1987; Vidhyasekaran et al., 1997; Yang et al., 2008). *R. solani* can be classified into at least 14 different anastomosis groups (AG-1 to AG-13 and a bridging isolate AG-BI) based on its morphological diversity, physiological diversity, host specificity and pathogenic diversity (Taheri and Tarighi, 2011). For instance, *R. solani* AG-3 PT is the main causal agent of potato black scurf which causes wilt and stalk rot on potato seedlings (Kuninaga et al., 1997, 2000). While *R. solani* AG-3 TB is the main pathogen of tobacco target spot, which induces necrosis and perforation lesions on the leaves that significantly reduced the economic quality of the plants (Lucas, 1975; Sneh et al., 1996; Gonzalez et al., 2001). *R. solani* AG-3 TB was first recorded in tobacco fields in the United States in the early 20th century (Lucas, 1975). This disease spread quickly and caused considerable losses nearly $ 20 million in Carolina (Shew and Main, 1985). In China, the tobacco target spot caused by *R. solani* AG-3 TB was first reported in 2006 in the tobacco fields of Liaoning province (Wu et al., 2012), and successively reported in Yunnan, Guangxi, and Sichuan province (Xu et al., 2018, 2021). The losses caused by the tobacco target spot are serious. In 2018, the tobacco yield in Gulin and Xuyong County of Luzhou, Sichuan Province was reduced by 20%, and the yield of serious fields was reduced by up to 90% (Xu et al., 2021). Due to its rapid transmission and genetic diversity, it is an urgent issue to clarify the pathogenesis of the fungus and explore effective disease resistance genes in the host plant.

Bioactive molecules such as toxins, enzymes and secreted proteins play important roles during *R. solani* infection (Yamamoto et al., 2019). Typical fungal pathogenic toxins such as succinic acid, PAA, furancarboxylic acid are isolated from *R. solani* AG-1IA, among which, PAA significantly inhibits the growth of roots of sugar beet (Aoki et al., 1963). A recent integrated study revealed that PAA and 3-Methylthiopropionic Acid (MTPA) produced by *R. solani* AG-3 PT, can cause degradation of the cell membrane, rough mitochondrial and cell walls, change of the shape of chloroplasts, and swollen endoplasmic reticulum (Kankam et al., 2016; Yamamoto et al., 2019). In addition to the toxins, enzymes involved in the production of secondary metabolites such as the non-ribosomal peptide synthases (NRPSs), polyketide synthases (PKSs), hybrid NRPS-PKS enzymes, prenyltransferases (DMATSs), and terpene cyclases (TCs) play the pathogenic role in fungi (Slot and Rokas, 2010). Moreover, a study demonstrated that the saprophytic nature of fungi has a close relation to their type and quantity of carbohydrate-active enzymes (CAZymes) (Cantarel et al., 2009). Currently, an array of CAZymes produced by *R. solani* was reported to degrade the cell wall of plants and express significantly during disease development (Lakshman et al., 2012). A total of 223 CAZymes and an expanded set of other cell wall degrading enzymes (CWDEs) genes, including those of *pectinase*, *xylanase* and *laccase* were secreted by *R. solani* AG-1 IA, which was associated with the pathogenicity and had a connection to the saprophytic lifestyle of fungi (Zheng et al., 2013). Furthermore, secreted proteins have been reported in many pathogenic fungi and play various roles in pathogenesis (Dutheil et al., 2016; Anderson et al., 2017; Fang et al., 2019). Some of the secreted proteins serve as ‘effectors’ that facilitate the infection of the pathogen as well as suppress host immunity responses (Dickman and de Figueiredo, 2013), while the reported numbers of the effectors differ between various fungi (Zheng et al., 2013; Anderson et al., 2017). A total of 1546 and 949 secretory proteins were predicted in *Magnaporthe grisea* and *A. laibachii*, respectively, and these proteins include the unusual carbohydrate-binding domains (Dean et al., 2005). In contrast, 965 secretory proteins have been predicted in *R. solani* AG-1 IA and most of their functions generally remain unclear (Zheng et al., 2013). Some of the effectors, such as AGLIP1, is a possible effector in *R. solani* AG-1 IA which inhibits basal defenses and promote disease development in plants (Li et al., 2019).

Until now, effective fungicides and highly resistant cultivars for *R. solani* are still very limited. Therefore, research in the molecular pathogenic mechanism of *R. solani* will provide valuable theoretical basis for disease control. Here, we analyzed the transcriptomes of *R. solani* AG-3 TB infecting leaves of *Nicotiana tabacum* at different time points, which were designated as early (6–12 h post inoculation, hpi), middle (24–36 hpi), and late (48–72 hpi) infection stage. The results of RNA-seq showed that several crucial genes involved in PAA synthesis of *R. solani* AG-3 TB were significantly increased, especially at the middle infection stage. And the expression of CAZymes and CWDEs genes gradually increased and peaked at the late infection stage. We also predicted 807 secretory proteins which may play a key pathogenic role during infection. These results provide extensive molecular basis for the pathogenic mechanisms of pathogen *R. solani* AG-3 TB during its infection in the host plants.

## Materials and methods

### *Rhizoctonia solani* AG-3 TB isolates and inoculation of tobacco

The *R. solani* AG-3 TB (YC-9) strain was isolated from severely infected tobacco plants in Kuandian County, Dandong City, Liaoning province of China (Wu et al., 2012). The YC-9 strain was activated in potato dextrose agar medium at 28°C for 3 days in the dark. The potato dextrose agar (PDA) medium with *R. solani* AG-3 TB (6 mm diameter) were inoculated on the acupuncture point and the cotton with sterile water was used for moisturizing. A time course study was performed by acupuncture inoculating *R. solani* YC-9 on the 5th and 6th leaf of tobacco variety Yunyan 87 (one of the commonly cultivated susceptible variety) at the 9th leaf stage, and harvested at 0, 6, 12, 24, 36, 48, 72 hpi. The center of the acupuncture part was taken, which was drilled with a 1.5 cm diameter punch, then the inoculated leaves were collected and frozen with liquid nitrogen. Four leaves were inoculated per tobacco and each leaf was inoculated four acupuncture points. A total of 105 tobacco plants were inoculated at 0, 6, 12, 24, 36, 48, 72 hpi, among which, 15 tobacco plants were inoculated at each time point and five of them were measured once as one biological replicate. Tobacco leaves inoculated with PDA medium serve at 0, 6, 12, 24, 36, 48, 72 hpi as mock treatment, the method of sample collection was the same as above. At the same time, the fungi were cultivated at PDA (0, 6, 12, 24, 36, 48, and 72 h), which were taken as the fungal control group for subsequent analysis.

### RNA extraction, library preparation, and sequencing

Total RNAs were extracted from the fungus inoculated leaf tissues at each time point using TRIzol Reagent (Invitrogen cat. NO.15596026). All the RNA samples were treated with DNase prior to mRNA isolation and sequencing, then the quality was determined using NanodropTM One Cspectrophotometer. And the 1.5% agarose gel electrophoresis using to determine the RNA integrity and using the Qubit 3.0 to quantify the final qualified RNAs. The total RNAs were subjected to stranded RNA sequencing library preparation. Each sample mentioned above was measured one time as one biological replicate. The generation sequencing library was constructed followed the Illumina’s recommendations. Oligo (dT) was used to purify poly (A)-containing mRNA from total RNA. Then the purified mRNA was fragmented and reverse transcribed to cDNAs. The short fragments were connected with adapters at both ends. Thereafter, the adaptor-ligated cDNA was performed using AxyPrep Mag PCR clean-up (Axygen) and recovered the fragments of ∼360 bp. The products were purified and enriched by PCR (11 cycles), and generated the indexed double-stranded cDNA library. The cDNA libraries were analyzed by Agilent 2100 Bioanalyzer and quantified by a Qubit 3.0 Fluorometer (Invitrogen, Carlsbad, CA, USA). Subsequently, the libraries were sequenced by paired-end sequencing under the platform of an Illumina HiSeq 6000 (SeqHealth Co., Ltd, Wuhan, China).

### RNA-seq data analysis and gene annotation

For transcriptomic analysis of *R. solani* AG-3 TB, raw sequencing data was first filtered by fastp (version 0.23.0) (Chen S. et al., 2018), low-quality reads were removed and the reads with adaptor sequences were trimmed. Then clean and deduplicated data were mapped to the reference genomes of *Nicotiana tabacum* from https://ftp.ncbi.nlm.nih.gov/genomes/all/GCF/000/715/135/GCF_000715135.1_Ntab-TN90/using STAR software (version 2.5.3a) with default parameters (Dobin et al., 2013) to remove the host transcripts. Then unmapped reads were *de novo* assembled by Trinity with the default parameters (Grabherr et al., 2011). Sequencing reads were mapped back to the assembled transcripts for assessing the quality of the transcriptome assembly using the Bowtie2 (Langmead and Salzberg, 2012). The longest transcripts of the same genes were screened as the unigenes for annotation and DEG analysis. For functional annotations of the unigenes, the protein databases Nr (NCBI non-redundant protein database), UniProt (universal protein database), Pfam (homologous protein family), eggNog (orthologous groups of genes), GO (Gene Onotology), and KEGG (Kyoto encyclopedia of genes and genomes) were used to infer the amino acid sequences.

### Differentially expressed gene analysis

The reads per kilobase per million mapped reads (RPKM) were used to compare the levels of differentially expressed genes (DEGs). Each sample in different time points (0, 6, 12, 24, 36, 48, and 72 hpi) was compared with the control. The tool of the EdgeR package (version 3.12.1) was utilized to identify the expression of DEGs (Robinson et al., 2010; McCarthy et al., 2012). The *p* value cut-off of 0.05 and a fold-change cut-off of 2 was used to determine the statistical significance of gene expression differences. The gene ontology (GO) analysis and Kyoto encyclopedia of genes and genomes (KEGG) enrichment for DEGs were analyzed by KOBAS software (version: 2.1.1) with a *p* value cut-off of 0.05 to determine the statistically significant enrichment (Wu et al., 2006).

### Secretory protein prediction

The screening of secretory proteins was performed based on the presence or absence of the predicted coding sequence of the signal peptide, transmembrane domain, ω-sites for glycosylphosphatidylinositol (GPI) anchor, transit peptides to mitochondrion and nuclear localization signal (Zheng et al., 2013). SignalP6.0^1^ was used to perform signal peptide cleavage site prediction. Transmembrane helices in the proteins were predicted using TMHMM.^2^ GPI-anchored proteins were identified using PredGPI.^3^ Proteins located in the mitochondria were determined by TargetP.^4^ Nuclear localization signal was predicted using NetNES.^5^ The proteins that comprise signal peptide cleavage sites and nuclear localization signal, helices without transmembrane domain together with the GPI-anchored proteins were retrieved as secreted proteins. Effector candidates were searched from among the predicted coding sequences of the transcriptome contigs using effectorP.^6^ Localization of probable effectors was predicted using the apoplastP.^7^

### Real-time quantitative PCR of candidate differentially expressed genes

To analyze the gene expression of the selected DEGs from each time point, Real-time quantitative PCR (qRT-PCR) was performed using a real-time PCR system Q711 (Vazyme Biotechnology, Nanjing, China) according to the manufacturer’s instruction. The quantitative PCR reaction was carried out in a 20 μl volume containing 1 μl of reverse transcription product, 10 μl of ChamQ Universal SYBR qRCR Master Mix, 0.4 μl of each primer (10 μM) and 8.2 μl of dd H_2_O. The reaction conditions for RT-qPCR including three steps (Step 1: 95°C, 30s, Reps1; Step 2: 95°C, 10s, 60°C, 30s, Reps40; Step 3: 60–95°C, increment 0.5°C/5s, Reps1). To verify gene expression of *R. solani* AG-3 TB during the growth and invasion stage, total cDNA was extracted from total RNA by time course (0, 6, 12, 24, 36, 48, and 72 hpi). The primers were designed by Primer Premier 5 (Supplementary Table 1).

## Results

### Tissue infection, transcriptome sequencing, *de novo* assembly and differentially expressed genes analysis

**FIGURE 1:**
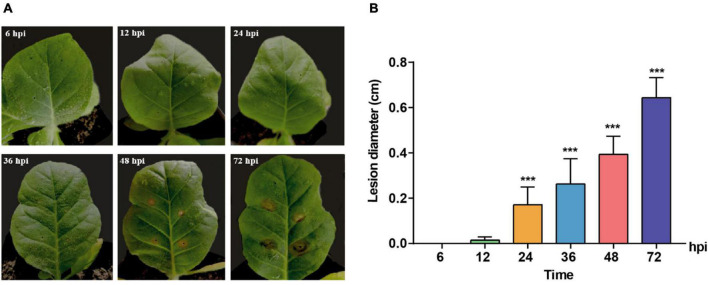
Symptoms change of *Nicotiana tabacum* inoculated with *R solani* AG-3 TB strain at different infection stages. **(A)** The symptoms on the Yunyan 87 leaf at 6, 12, 24, 36, 48, 72 hours post inoculation (hpi), respectively. **(B)** Measurement of the lesion diameter (cm) at 6 (control), 12, 24, 36, 48, 72 hpi. The asterisks show the statistical significances using the two-tailed *t*-test (*p < 0.05, **p < 0.01, ***p < 0.001).

A time course observation and sampling were conducted to clarify the induction of host symptoms and gene expression of pathogenic fungus *R. solani* AG-3 TB at different infection stages. The results indicated that tobacco leaves with pathogen did not show observable symptoms at 6 and 12 hpi, while the obvious yellow halo can be observed at the inoculated site at 24 hpi. The symptom aggravated with the appearance of wheel pattern after 72 hpi (Figure 1A). The diameters of the necrotic lesions were 0.0036, 0.0152, 0.1709, 0.2633, 0.3939, 0.6434 cm at 6, 12, 24, 36, 48, 72 hpi, respectively (Figure 1B).

Thereafter, an integrated transcriptomic analysis was conducted to globally reveal the crucial genes and pathways involved in *R. solani* AG-3 TB infection on *N. tabacum* at different stages, specifically at 6, 12, 24, 36, 48, and 72h after pathogen inoculation. The assembly statistics of *R. solani* AG-3 TB infection showed a total of 35,415,039 contigs, lengths of N50 and N90 with 1487 and 321 bp, respectively, with 49.06% GC content, of which the GC content maximum was 87.07% and the GC content minimum was 22.52% (Table 1).

**TABLE 1:** Data statistics of the transcriptome sequencing in *R solani* AG-3 TB subgroups.

Type	Trinity	Unigene
N50	2126	1487
N90	554	321
average length	1293.52	826.3
Max length	17497	17497
Min length	201	201
Total base	108235063	35415039
Total contigs	83675	42860
GC content (%)	49.36	49.06
GC content max	87.07	87.07
GC content min	22.52	22.52

The six time points were designated as the early stage (6–12 hpi), middle stage (24–36 hpi), and late stage (48–72 hpi) after *R. solani* AG-3 TB inoculation. The DEGs change showed that 37,999 DEGs were detected in the early stage, including 18,317 up-regulated genes and 19,682 down-regulated genes after *R. solani* AG3 TB inoculation. In contrast, the number of DEGs were 43,371, including 28,133 up-regulated and 15,238 down-regulated genes in the middle stage, which comprised largest amounts of DEGs (Supplementary Figure 1 and Supplementary Table 2). These results suggested that the number change of DEGs have the difference during in various hours post inoculation, which may be related to pathogenic factors secreted of *R. solani* AG-3 TB.

### Enrichment analysis of differential gene pathway

Functional enrichment analysis is an important way to retrieve some significant DEGs for organisms. The Gene Ontology related to three items including biological processes, molecular function and cellular components was analyzed. The results showed that DEGs involved in biological processes including the fatty acid metabolism process, pyridine-containing compound metabolic process and cellular components including proton-transporting V-type ATPase complex and cytosolic were significantly enriched compared with other items in the early infection stage of *R. solani* AG-3 TB (6–12 hpi) (Supplementary Figure 2A). At 24 hpi after the fungus inoculation, the critical items such as biological processes involved in cellular carbohydrate biosynthetic process, response to stress were enriched, and cell periphery related to cellular components was enriched (Supplementary Figure 2B). In addition, the MAPK cascades involved in biological processes was significantly enriched in the late stage of infection (48 –72 hpi) (Supplementary Figure 2C).

**FIGURE 2:**
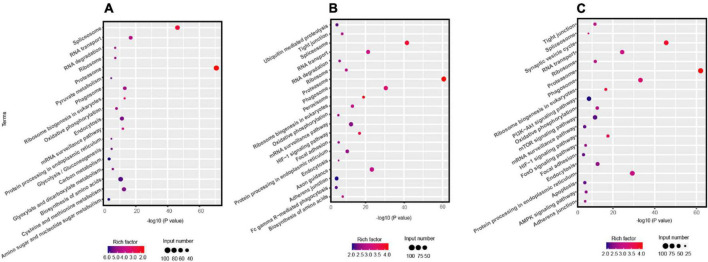
KEGG pathway analysis the DEGs of *R solani* AG-3 TB at different stage. **(A)** KEGG pathway analysis at 6 hpi stage. **(B)** KEGG pathway analysis at 24 hpi stage. **(C)** KEGG pathway analysis at 72 hpi stage.

The KEGG pathway analysis indicated that the enrichment pathways of *R. solani* AG-3 TB were diverse in different infection stages. The results showed that amino sugar and nucleotide sugar metabolism, carbon metabolism, biosynthesis of amino acids and other pathway were significantly enriched in the early infection stage (6 –12 hpi) (Figure 2A). In contrast, the ubiquitin mediated proteolysis pathway was the most significantly enriched item in the middle stage (Figure 2B). Specifically, pyrimidine metabolism pathway was only enriched in the middle infection stage (24 hpi). Furthermore, the amino sugar and nucleotide sugar metabolism, and biosynthesis of amino acid pathways were enriched, while the carbon metabolism pathway was not enriched at 72 hpi stage (Figure 2C).

### Gene expression involved in the biosynthesis of fungal toxin

**FIGURE 3:**
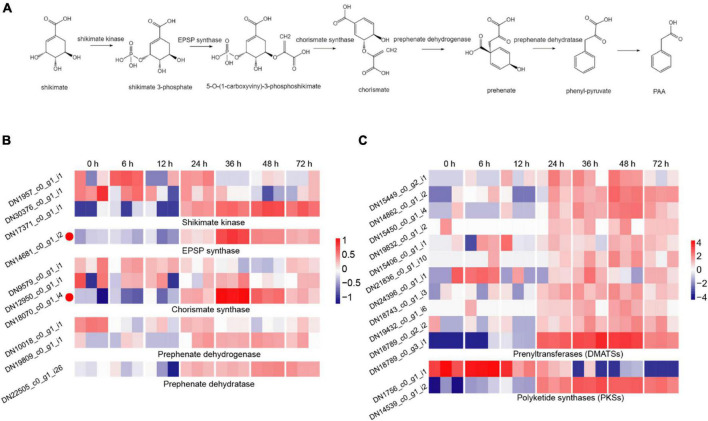
The DEGs for secondary metabolites production. **(A)** The biosynthetic pathway for PAA production, including the five key synthetases (shikimate kinase, EPSP synthase, chorismate synthase, prephenate dehydrogenase, and prephenate dehydratase) of PAA and six synthetic compounds associated with shikimate 3-phosphate, 5-O-(1-carboxyviny)-3-phosphoshikimate, chorismite, prehenate, phenyl-pyruvate, PAA. **(B)** The expression patterns of candidate genes on the five key synthetases of PAA biosynthetic pathway. The heatmap describes the expression of candidate genes (RPKM in log2-scale) associated with shikimate kinase, EPSP synthase, chorismate synthase, prephenate dehydrogenase, and prephenate dehydratase. The red circles next to the heatmap were candidate genes tested by qRT-PCR. **(C)** The expression patterns of candidate genes on DMATSs and PKSs. The heatmap describes candidate gene expression of DMATSs and PKSs.

The synthesis of fungal toxin PAA requires five important enzymes, including shikimate kinase, 3-phosphoshikimate 1-carboxyvinyltransferase (EPSP synthase), chorismate synthase, prephenate dehydrogenase and prephenate dehydratase, of which, shikimate is used as the initial material and the phenyl-pyruvate is the precursor of PAA (Cook et al., 2016; Figure 3A). In the DEGs during *R. solani* AG-3TB infection, the critical enzymes required for PAA synthesis were retrieved, and their expression levels were gradually increased in different infection stages. A total of 10 DEGs were selected and the expression levels of chorismate synthase, prephenate dehydrogenase were increased in the early and middle stage, while those of shikimate kinase, EPSP synthase and prephenate dehydratase were up-regulated in the middle and late stage of infection (Figure 3B). Specifically, the expression of five enzyme genes in PAA synthesis pathway increased to the highest levels in the middle infection (24 hpi).

Prenyltransferases (DMATSs) and polyketide synthases (PKSs) are two important enzymes involved in the production of secondary metabolites (Slot and Rokas, 2010). The number of DMATSs and PKSs were 11 and 2, and those DMATSs and PKSs genes expression continuously increased from 24 to 48 hpi stage (Figure 3C). The expression levels of most DMATSs were increased at 24 hpi and peaked at 48 hpi. As for PKSs, the expression of DN1756_c0_g1_i1 was increased in the early stage, while the expression of DN14539_c0_g1_i2 increased significantly at 48 hpi (Figure 3C).

### Gene expression of *Rhizoctonia solani* AG-3 pathogenic related enzymes

**FIGURE 4:**
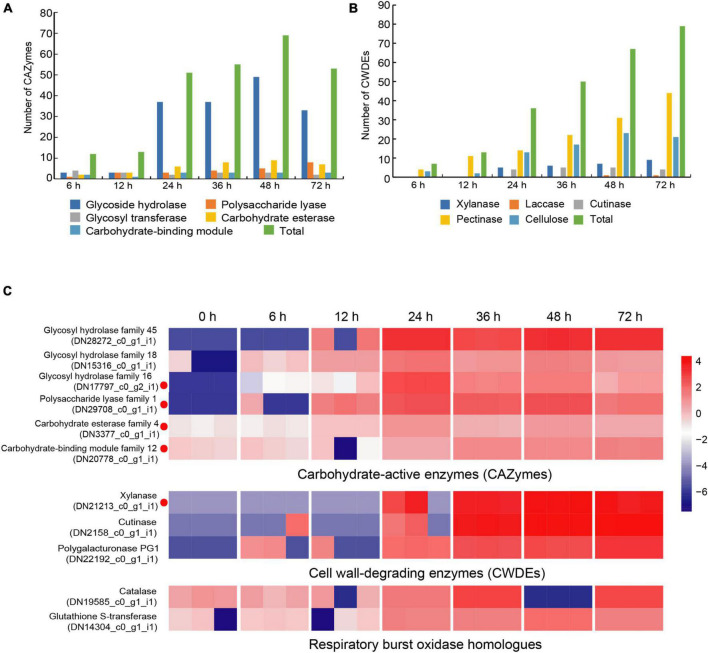
Changes of different enzyme genes during various infection stages. **(A)** The number of CAZymes at different infection stage. **(B)** The number of CWDEs at different infection stages. **(C)** Expression patterns of candidate genes on CAZymes (*glycosy hydrolase family 45*, *glycosy hydrolase family 18*, *glycosy hydrolase family 16*, *polysaccharide lysae family 1*, *carbohydrate esterase family 4*, *carbohydrate-binding module family 12*), CWDEs (*xylanase*, *cutinase*, *polygalacturonase PG1*) and respiratory burst oxidase homologs (*catalase*, *glutathione S-transferase*). The heatmap describes the enzyme gene expression at different stages (RPKM in log10-scale). The red circles next to the heatmap were candidate genes tested by qRT-PCR.

**FIGURE 5:**
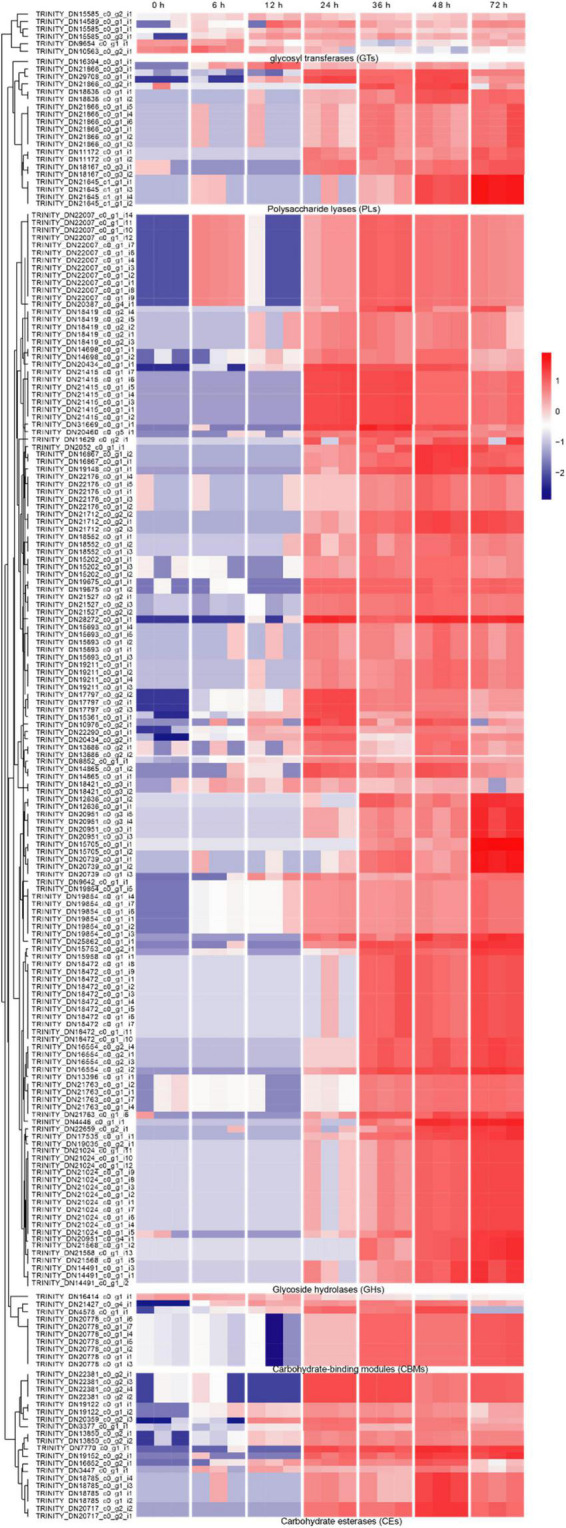
The heatmap of the expression levels of CAZymes genes in different stages expression patterns of candidate genes (RPKM in log10-scale) for CAZymes were represented in heatmaps (numerals indicate hours after inoculation onto tobacco).

Carbohydrate-active enzymes (CAZymes) is a large gene family involved in the construction and breakdown of complex carbohydrates and glycoconjugates, and mainly comprise Glycoside hydrolases (GHs), Glycosyl transferases (GTs), Polysaccharide lyases (PLs), Carbohydrate esterases (CEs) and Carbohydrate-binding modules (CBMs) (Cantarel et al., 2009). The transcriptomic results showed that the gene expression levels of 12, 13, 51, 55, 69, and 53 CAZyme families were gradually up-regulated from the 6 to 72 hpi (Figure 4A). In the early infection stage (6–12 hpi), the secretion of CAZymes was low. Then, the number of CAZymes related DEGs rapidly increased at 24 hpi and peaked at 48 hpi (Figure 5). The quantities of *GHs* and *PLs* were slightly higher than other components during *R. solani* AG-3 TB infection. Results of the heatmap indicated that expression levels of *GH45*, *GH18* and *GH16* genes were progressively increased in the early infection stage, while those of *PL1*, *CE4* and *CBM12* genes were increased in the middle and late infection stages, respectively (Figure 4C).

Cell wall-degrading enzymes (CWDEs) produced by plant pathogenic fungi, especially those without special penetration, can damage the cell wall polymers (Kubicek et al., 2014). Among these fungi, *R. solani* can produce CWDEs including pectinase, xylanase, laccase, cutinase and cellulase (Zheng et al., 2013). Here, the pectinase, xylanase, laccase, cutinase and cellulase of *R. solani* AG-3 TB were retrieved from the RNA-seq (Supplementary Figure 3). The result indicated that expression of 7 CWDEs genes (pectinase and cellulose) were increased in the initial stage of infection (6 hpi), while 79 total genes including pectinase, xylanase, laccase, cutinase and cellulase were significantly increased at 72 hpi (Figure 4B). According to the expression levels of CWDEs, *xylanase* (TRINITY_DN21213_c0_g1_i1) and *cutinase* (TRINITY_DN2158_c0_g1_i1) were increased at 24 hpi, while those of the *PG1* (TRINITY_DN22192_c0_g1_i1) were up-regulated at 6 hpi (Figure 4C).

When pathogenic fungi infect plants, the respiratory burst oxidase homologs play an important role to increase their pathogenicity (Ghosh et al., 2014). To clarify the gene change involved in respiratory burst oxidase homologs during the interaction between *R. solani* AG-3 TB and tobacco, the catalase, glutaredoxin, glutathione peroxidase, glutathione S-transferase, copper/zinc superoxide dismutase, and iron/manganese superoxide dismutase were retrieved for further analysis. The results showed that the expression of respiratory burst oxidase homologs increased exponentially in the middle and late stages of infection (Supplementary Figure 4). Additionally, the expression of *catalase* (TRINITY_DN19585_c0_g1) was significantly up-regulated in the middle stage of infection (36 hpi), and the expression of *glutathione S-transferase* (TRINITY_DN14304_c0_g1) was increased in the late stage of infection (48 hpi) (Figure 4C).

### The gene sequences of *Rhizoctonia solani* AG-3 TB for the secretomes

**FIGURE 6:**
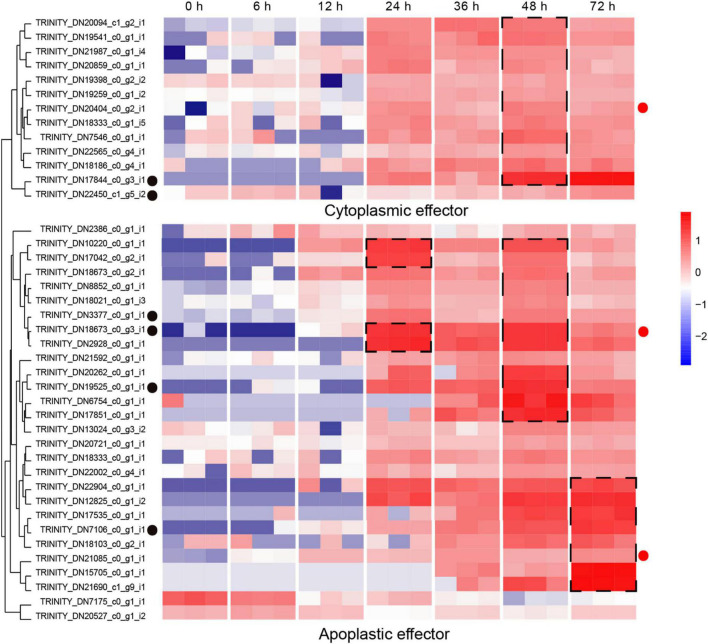
Expression patterns of candidate apoplastic and cytoplasmic effectors The heatmap represents apoplastic and cytoplasmic effectors genes expression (RPKM in log10-scale). The genes retrieved from Pfam, Blast and reads counts (>50). The up-regulated clusters were showed in dot black boxes. The red circles next to the heatmap were candidate genes tested by qRT-PCR.

The secretion of biologically active proteins is a fundamental infection strategy during the interaction between plants and fungi (Lo Presti et al., 2015). Secretomes of fungi play important roles in infection, colonization and pathogenicity (Xia et al., 2020). We adopted the classical secretion pathway (searching protein domains) as well as the apoplastic and cytoplasmic effectors (Effectrop 2.050 and ApoplastP) to retrieve the secreted proteases during the *R. solani* AG-3 TB infection. A total of 807 potential secretomes were retrieved, and 124 apoplastic effectors and 236 cytoplasmic effectors were predicted. There are few reports on apoplastic and cytoplasmic effectors of fungus, but some report had revealed that cytoplasmic effectors in *phytophthora sojae* encoding conservative sequences such as *RxLR* can cause tissue necrosis of plants (Zhang et al., 2015; Sperschneider et al., 2018). A total of 13 cytoplasmic effectors and 28 apoplastic effectors were retrieved, and most of these effectors can be classified in to serine protease (TRINITY_DN17844_c0_g3_i1), eukaryotic metallothionein (TRINITY_DN22450_c1_g5_i2), polysaccharide deacetylase (TRINITY_DN3377_c0_g1_i1), acetylxylan esterase (TRINI TY_DN7106_c0_g1_i1), extracellular metalloproteinases (TRINITY_DN18673_c0_g3_i1), and deuterolysin metalloprotease (TRINITY_DN19525_c0_g1_i1) families (Supplementary Table 3). The expression analysis of pathogenic protease genes showed that the candidate cytoplasmic effector genes were significantly up-regulated at the late infection stage (48 hpi), while the genes of apoplastic effector were enriched significantly in the middle and late infection stages (24, 48 hpi) (Figure 6).

Small cysteine-rich proteins play functional roles in the molecular interaction between fungi and plant (Stergiopoulos and de Wit, 2009), and generally have a classical structural characteristic with a less than 300aa protein and more than 4% cysteine (Yamamoto et al., 2019). Here, a total of 78 potential small cysteine-rich proteins were retrieved from *R. solani* AG-3 TB data (Supplementary Table 4). It should be noted that most of the small cysteine-rich proteins are unnamed protein products, which still require further investigation for clarification of their functions.

### qPCR verification of transcriptome up-regulated genes

**FIGURE 7:**
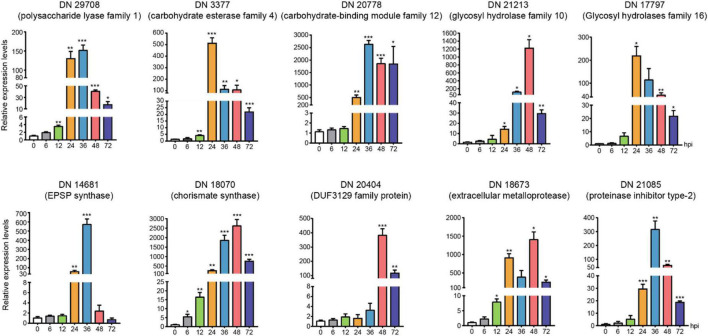
The expression levels of candidate genes expression patterns of candidate genes associated with toxins, enzymes, and secreted proteins using quantitative real time RT-PCR verification at 0, 6(control), 12, 24, 36, 48, and 72 hours after inoculation. The asterisks showed the statistical significances using the two-tailed t-test (*p < 0.05, **p < 0.01 ***p < 0.001).

We chose the DEGs with large difference expression and revealed the potential critical roles (CAZymes, toxins and effectors) during *R. solani* AG-3 TB infection in different infection stages (6 hpi, 12 hpi, 24 hpi, 36 hpi, 48 hpi, 72 hpi). The expression levels of DN3377 (TRINITY_DN3377_c0_g1_i1), DN14681 (TRINITY_DN14681_c0_g1_i2), DN17797 (TRINI TY_DN17797_c0_g2_i1), DN18673 (TRINITY_DN18673_c0 _g3_i1), DN20404 (TRINITY_DN20404_c0_g2_i1), DN20778 (TRINITY_DN20778_c0_g1_i1), DN29708 (TRINITY_DN2 9708_c0_g1_i1), DN21213 (TRINITY_DN21213_c0_g1_i1), DN18070 (TRINITY_DN18070_c0_g1_i4), DN21085(TRIN ITY_DN21085_c0_g1_i1) were verified by qRT-PCR. The result indicated that CAZymes (DN29708, DN3377, DN20778, DN21213, and DN17797) expression were diverse, but increased levels were found for those five genes in the middle and late infection stages. EPSP synthase (DN14681) and chorismite synthase (DN18070) were two important genes involved in PAA synthesis pathway, and their expression started increasing at 24 hpi. The 36 hpi and 48hpi were important infection stages for *secretory protein* (DN20404, DN18673, and DN21085). These results indicated that the pathogenic genes expression obviously changed and also proved the reliability of transcriptomics during *R. solani* AG-3 TB infection (Figure 7).

## Discussion

*Rhizoctonia solani* is an important group of saprophytic soilborne basidiomycetes that causes significant losses to a variety of crops. The complex multiple AGs and multi-nuclear nature of *R. solani* make it difficult to thoroughly understand its pathogenesis and development mechanism. Therefore, clarification of gene expression patterns in the development and infection of *R. solani* is crucial for the following research on the pathogenic mechanism and effective control of the fungus.

Before doing transcriptomic analysis, the AG3-T5^8^ (Kaushik et al., 2020) as the reference genomes was mapped with the sequencing data of AG-3 TB. But the mapped rates of reads were low (<41%) (Supplementary Table 5). The low mapping rates may suggest that the reported strains with genetic data is quite distinct from AG-3 TB. Therefore, we used the transcriptome assembly from RNA-Seq data without reference genome. And according to the way of RNA-Seq data analysis without a reference genome, the quantity of DEGs was large than other *R. solani*. The reason for this result related to the fragmented genes and isoform genes were produced caused an increase in the number of DEGs.

In this study, many lines of critical DEGs in different stages of *R. solani* AG-3 TB infection were investigated, and showed various enrichment of the pathways. In the early infection stage, fatty acid metabolism, amino sugar, nucleotide sugar metabolism, carbon metabolism and biosynthesis of amino acids were significantly enriched. Such metabolisms have been indicated to correlate with branching, initiation and elongation, cell wall and biofilm matrix of fungal hyphae (Chen et al., 2020; Liboro et al., 2021). In the middle stage of infection, the cellular carbohydrate biosynthetic process of *R. solani* AG-3 TB began to be enriched. One gene in cellular carbohydrate biosynthetic process was predicted as *trehalose-phosphate phosphatase* (TRINITY_DN20403_c0_g1), and it associated with the development of sclerotia, stress response, and protection of cells from hydrogen peroxide in some pathogenic fungi (Jiang et al., 2017; Wang et al., 2018). Furthermore, ubiquitin-proteasome mediated protein turnover and the pyrimidine metabolic pathway was significantly enriched in the middle stage, which have a close relation to stress response, host adaptation and fungal pathogenesis (Qin et al., 2020). Herein, the detailed results of DEGs variation and pathway analysis indicated that mycelial growth and development should occur in the early stage of infection (6–12 hpi), while the crucial pathogenic stage of *R. solani* AG-3 TB may occur in the middle and late stages of infection.

Phenylacetic acid is an organic compound that can be produced by many kinds of fungi with various functions (Moore and Towers, 1967; Moore et al., 1968; Siddiqui and Shaukat, 2005). For example, PAA is the side chain precursor in the biosynthesis of penicillin (Mohammad-Saeid et al., 2018), and the catabolism of PAA is closely related to the virulence of *Burkholderia cenocepacia* (Lightly et al., 2019). PAA was also indicated to be an important signal molecule during microbial interactions with their hosts (Lightly et al., 2017). In a previous study, we isolated and purified a PAA derivative, namely 3-methoxyphenylacetic acid (C_9_H_10_O_3_) from *R. solani* AG-3 TB, and confirmed its structure using thin layer chromatography (TLC), high performance liquid chromatography (HPLC), IR and NMR spectra (Hou, 2018). Importantly, five enzymes including shikimate kinase, EPSP synthase, chorismate synthase, prephenate dehydrogenase and prephenate dehydratase were considered to play crucial roles in the synthesis of PAA (Cook et al., 2016). Among those genes, prephenate dehydrogenase is an important enzyme associated with virulence and defense in fungi (Lopez-Nieves et al., 2019). A study showed that the expression of *prephenate dehydratase* of *R. solani* AG-1 IA was markedly increased at 18 h after infection (Zheng et al., 2013). In this study, we investigated the time-course expression of the five genes involved in PAA synthesis of *R. solani* AG-3 TB, and noted most of these genes were rapidly up-regulated at 24 hpi, which may be a critical time point for toxin production of the fungus. While precise detection of PAA at each infection stage should be conducted in the following study to clarify this hypothesis. In addition, many lines of secondary metabolites may also play roles in *R. solani* AG-3 TB infection. We herein investigated several ‘backbone’ enzymes for the synthesis of secondary metabolites from *R. solani* AG-3 TB, including prenyltransferases (DMATSs) and polyketide synthases (PKSs) (Slot and Rokas, 2010). Studies have indicated that DMATSs are involved in the production and secretion of indole alkaloids secondary metabolites (Julia et al., 2016; Arndt et al., 2017), while the PKSs are required for pigment production in fungi, which have a central role in the pathogenicity of fungi (Chen X. et al., 2018; Liu et al., 2021). In this study, our results showed that expression levels of DMATSs and PKSs genes significantly increased in the early and middle infection stage. These results collectively suggested that *R. solani* AG-3 TB may produce secondary metabolite classes or complex compounds to damage the plant or play the function of parasite life cycle.

Cell wall degrading enzymes (CWDEs) secreted by pathogenic fungi are advantageous to the colonization, expansion and spread of fungi. Furthermore, the amount and species of CWDEs produced by the pathogenic fungi during infection differ between monocot or dicot host plants (Cuomo et al., 2007; King et al., 2011). The fungus *Macrophomina phaseolina* was reported to secrete 49 kinds of CWDEs involved in cellulose and homogalacturonan degradation when infecting sorghum (Bandara et al., 2018). In the study of genome analysis interaction between *R. solani* AG-1 IA and rice, the pectinase genes, xylanase genes, and laccase genes can be produced by *R. solani* AG-1 IA, among which the laccase genes, pectinase genes may play specific roles to necrotrophic life cycle (Zheng et al., 2013). In addition, treatment *in vitro* expressed pectinase PG2 of *R. solani* AG-1 IA can cause necrosis symptoms in the rice tissue (Chen et al., 2020). In the previous study, we have shown that the pectinase (PG, PMG, PGTE, and PMTE) and cellulase (Cx, β-Glucosidase) of *R. solani* AG-3 TB have the highest activity in the culture medium of Marcus in 18 hpi during *R.solani* AG-3 TB infection (Fu, 2011). In this study, the results of RNA-seq and qRT-PCR demonstrated that expression levels of 7 CWDEs in *R. solani* AG-3 TB were increased in 6 hpi, while those of 79 CWDEs (pectinase, xylanase, laccase, cutinase, and cellulase) were significantly up-regulated in 48-72 hpi. These results systemically investigated the expression of these critical CWDEs at each infection stages, and suggested their possible functions in the pathogenesis of *R. solani* AG-3 TB as well as the induction of necrotic symptoms of the host.

Moreover, we also found that respiratory burst oxidase homologs genes including catalase, glutaredoxin, glutathione peroxidase, glutathione S-transferase, copper/zinc superoxide dismutase, and iron/manganese superoxide dismutase were differentially regulated in the transcriptome of *R. solani* AG-3 TB infection. The respiratory burst oxidase homologs were reported to detoxify ROS produced by plant (Ghosh et al., 2014). Studies have shown that the fungal respiratory burst oxidase homologs were involved in the colonization of necrotrophic fungi as well as the induction of host necrotic symptoms (Kámán-Tóth et al., 2018). An RNA-seq study indicated that the expression levels of two respiratory burst oxidase homologs genes of *R. solani* AG-1 IA were increased during ROS production (Zhang et al., 2017), and the oxidases were reported to be associated with the colonization of this fungus (Pauly, 2012). In this study, the expressions of respiratory burst oxidase homologs genes were increased in the middle and late infection stage, which indicated that these critical genes may be associated with the detoxification and oxidative stress for the pathogen of tobacco target spot.

Generally, pathogens can produce many kinds of effectors that are particularly important in promoting pathogen expansion and inhibiting host defense (Giraldo and Valent, 2013; Lo Presti et al., 2015). *R. solani* has become one of the most devastating plant fungal pathogens in the past decade and it is difficult to clarify its genetic characteristics and pathogenicity because of anastomosis groups and multinuclear nature (Yamamoto et al., 2019). During genomic analysis of *R. solani* AG-1 IA, a total of 965 secreted proteins were predicted, including 103 potential small cysteine-rich proteins (Zheng et al., 2013). In contrast, little is known about the secreted proteins as well as the effectors produced by *R. solani* AG-3 TB. In this study, 807 possible secretory proteins were predicted from *R. solani* AG-3 TB, which comprise possible 124 apoplastic effectors and 236 cytoplasmic effectors. In addition, 41 genes were predicted as deuterolysin metalloprotease, serine protease, and extracellular metalloproteinases. The fungal deuterolysin metalloprotease (M35) family was reported to be involved in cell wall degradation, epidermal growth inhibition, and cell activity of insects (Huang et al., 2020). The serine protease is an important pathogenic marker for *Alternaria solani* (Chandrasekaran et al., 2014). We presumed that these deduced secreted proteins in pathogen of tobacco target spot may serve as potential effectors that may play important roles in the pathogenicity of fungus, and remains to be further investigated in the following work.

In this study, we conducted integrated transcriptomic analysis and revealed many lines of potentially critical genes involved in the pathogenesis of *R. solani* AG-3 TB on *Nicotiana tabacum* at different infection stages. The results showed that various enzymes, toxins as well as effectors may play different, but critical roles in the interaction between pathogen and plant. Based on the results of systemic analysis of RNA-seq, we proposed the pathogenic mechanisms of *R. solani* AG-3 TB infecting plants. The hypha of *R. solani* AG-3 TB should begin to develop and grow in the leaves during the early infection stage (6–12 hpi). Then, the critical toxins and effectors may synergistically suppress plant defense response and regulate the infection of *R. solani* AG-3 TB in the middle stage (24–36 hpi). At the late stage (48–72 hpi), the plant cell structure and tissue were continuously eroded by toxins and CWDEs, which resulted in necrosis in leaves (Supplementary Figure 5). These results collectively provide critical insights into many lines of potentially functional genes as well as the pathways involved in the pathogenesis of tobacco target spot, and provide valuable theoretical basis for the accurate prevention and control of the disease. This is the first time to predict potentially functional genes for AG-3 TB, the agent of tobacco target spot by the transcriptome analyses. According to our results, the functional genes between AG-1 IA and AG-3 TB have big difference, for instance, the number of enzymes and effectors in AG-1 IA is larger than AG-3 TB (Zheng et al., 2013). Moreover, the PAA is a potential pathogenicity toxin in AG-1 IA, AG-3 TB, and AG-3 PT (Kankam et al., 2016; Yamamoto et al., 2019). Therefore, comparison the difference in candidate effectors or toxin genes from different AG strains will be an important aspect to investigate the genetic characteristics and pathogenic differences.

## Data availability statement

The datasets presented in this study can be found in online repositories. The names of the repository/repositories and accession number(s) can be found below: https://www.ncbi.nlm.nih.gov/, PRJNA853492.

## Author contributions

CZ and YW: conceptualization and project administration. XL, CX, LJ, FY, and YY: methodology and software. XL: formal analysis and data curation. XL and MA: resources and writing—original draft preparation. YW: supervision and funding acquisition. All authors read and agreed to the published version of the manuscript.

## Footnotes

1
https://services.healthtech.dtu.dk/service.php?SignalP-6.0

2
http://www.cbs.dtu.dk/services/TMHMM-2.0/

3
http://gpcr.biocomp.unibo.it/predgpi/pred.htm

4
https://services.healthtech.dtu.dk/service.php?TargetP-2.0

5
https://services.healthtech.dtu.dk/service.php?NetNES-1.1

6
http://effectorp.csiro.au/

7
http://apoplastp.csiro.au/data.html

8
https://ftp.ncbi.nlm.nih.gov/genomes/all/GCA/905/219/615/GCA_905219615.1_AG3-T5/

## References

[B1] Anderson J. P. Sperschneider J. Win J. Kidd B. Yoshida K. Hane J. (2017). Comparative secretome analysis of *Rhizoctonia solani* isolates with different host ranges reveals unique secretomes and cell death inducing effectors. *Sci. Rep.* 7:10410. 10.1038/s41598-017-10405-y 28874693 PMC5585356

[B2] Aoki H. Sassa T. Tamura T. (1963). Phytotoxic Metabolites of *Rhizoctonia solani*. *Nature* 20 575–575. 10.1038/200575a0

[B3] Arndt B. Janevska S. Schmid R. Hübner F. Humpf H. U. (2017). A fungal N-dimethylallyltryptophan metabolite from *Fusarium fujikuroi*. *Communication* 18 899–904. 10.1002/cbic.201600691 28295904

[B4] Bandara Y. M. A. Y. Weerasooriya D. K. Liu S. Little C. R. (2018). The necrotrophic fungus *Macrophomina phaseolina* promotes charcoal rot susceptibility in grain sorghum through induced host cell wall-degrading enzymes. *Phytopathology* 108 948–956. 10.1094/PHYTO-12-17-0404-R 29465007

[B5] Cantarel B. L. Coutinho P. M. Corinne R. Thomas B. Vincent L. Bernard H. (2009). The Carbohydrate-Active EnZymes database (CAZy): An expert resource for Glycogenomics. *Nucleic Acids Res.* 37 233–238. 10.1093/nar/gkn663 18838391 PMC2686590

[B6] Chandrasekaran M. Chandrasekar R. Sa T. Sathiyabama M. (2014). Serine protease identification (*in vitro*) and molecular structure predictions (*in silico*) from a phytopathogenic fungus, *Alternaria solani*. *J. Basic Microbiol.* 54(Suppl. 1), S210–S218. 10.1002/jobm.201300433 24122785

[B7] Chen S. Zhou Y. Chen Y. Gu J. (2018). fastp: An ultra-fast all-in-one FASTQ preprocessor. *Bioinformatics* 34 884–890. 10.1101/274100 30423086 PMC6129281

[B8] Chen X. Meng X. Cheng F. Hu C. (2018). Progress in fungal polyketide biosynthesis. *Chin. J. Biotechnol.* 34 151–164. 10.13345/j.cjb.170219 29424130

[B9] Chen Y. Mauff F. L. Wang Y. Lu R. Zhang S. (2020). The transcription factor soma synchronously regulates biofilm formation and cell wall homeostasis in *Aspergillus fumigatus*. *mBio* 11:e02329-20. 10.1128/mBio.02329-20 33173002 PMC7667024

[B10] Cook S. D. Nichols D. S. Smith J. Chourey P. S. Ross J. J. (2016). Auxin biosynthesis: Are the Indole-3-acetic acid and phenylacetic acid biosynthesis pathways mirror images? *Plant Physiol.* 171 1230–1241. 10.1104/pp.16.00454 27208245 PMC4902625

[B11] Cuomo C. A. Guldener U. Xu J. R. Trail F. Turgeon B. G. (2007). The *Fusarium graminearum* genome reveals a link between localized polymorphism and pathogen specialization. *Science* 317 1400–1402. 10.1126/science.1143708 17823352

[B12] Dean R. A. Talbot N. J. Ebbole D. J. Farman M. L. Mitchell T. K. Orbach M. J. (2005). The genome sequence of the rice blast fungus *Magnaporthe grisea*. *Nature* 434 980–986. 10.1038/nature03449 15846337

[B13] Dickman M. B. de Figueiredo P. (2013). Death be not proud-cell death control in plant fungal interactions. *PLoS Pathog.* 9:e1003542. 10.1371/journal.ppat.1003542 24068920 PMC3771904

[B14] Dobin A. Davis C. A. Schlesinger F. (2013). STAR: Ultrafast universal RNA-seq aligner. *Bioinformatics* 29 15–21. 10.1093/bioinformatics/bts635 23104886 PMC3530905

[B15] Dutheil J. Y. Mannhaupt G. Schweizer G. Sieber C. M. K. Münsterkötter M. Güldener U. (2016). A tale of genome compartmentalization: The evolution of virulence clusters in smut fungi. *Genome Biol. Evol.* 8 681–704. 10.1093/gbe/evw026 26872771 PMC4824034

[B16] Fang A. Gao H. Zhang N. Zheng X. Sun X. (2019). A novel effector gene *SCRE2* contributes to full virulence of *Ustilaginoidea virens* to Rice. *Front. Microbiol.* 10:845. 10.3389/fmicb.2019.00845 31105658 PMC6492501

[B17] Fu Y. (2011). *Study on the genetic differentiation, infection characteristics and pathogenic mechanism of Rhizoctonia solani.* Shenyang: Shenyang Agriculture University.

[B18] Ghosh S. Gupta S. K. Jha G. (2014). Identifcation and functional analysis of AG1-IA specifc genes of *Rhizoctonia solani*. *Curr. Genet.* 60 327–341. 10.1007/s00294-014-0438-x 25070039

[B19] Giraldo M. C. Valent B. (2013). Filamentous plant pathogen effectors in action. *Nat. Rev. Microbiol.* 11 800–814. 10.1038/nrmicro3119 24129511

[B20] Gonzalez D. Carling D. E. Kuninaga S. Vilgalys R. Cubeta M. A. (2001). Ribosomal DNA systematics of *Ceratobasidium* and *Thanatephorus* with *Rhizoctonia anamorphs*. *Mycologia* 93:1138. 10.2307/3761674

[B21] Grabherr M. G. Haas B. J. Yassour M. Levin J. Z. Thompson D. A. Amit I. (2011). Full-length transcriptome assembly from RNA-Seq data without a reference genome. *Nat. Biotechnol*. 29 644–652. 10.1038/nbt.1883 21572440 PMC3571712

[B22] Hou H. H. (2018). *The active components and their pathogenic mechanism of toxins produced by Rhizoctonia solani from tobacco target spot disease.* Shenyang: Shenyang Agriculture University.

[B23] Huang A. Lu M. Ling E. Li P. Wang C. S. (2020). A M35 family metalloprotease is required for fungal virulence against insects by inactivating host prophenoloxidases and beyond. *Virulence* 11 222–237. 10.1080/21505594.2020.1731126 32079481 PMC7051145

[B24] Jiang H. Liu G. L. Chi Z. Hu Z. Chi Z. M. (2017). Genetics of trehalose biosynthesis in desert-derived *Aureobasidium melanogenum* and role of trehalose in the adaptation of the yeast to extreme environments. *Curr. Genet.* 64 479–491. 10.1007/s00294-017-0762-z 29018921

[B25] Julia W. Xie X. L. Li S. M. (2016). Characterisation of 6-DMATS_(Mo) from *Micromonospora olivasterospora* leading to identification of the divergence in enantio-selectivity, regioselectivity and multiple prenyla-tion of tryptophan prenyltransferases. *Org. Biomol. Chem.* 14, 9883–9895. 10.1039/c6ob01803c 27714299

[B26] Kámán-Tóth E. Dankó T. Gullner G. Bozsó Z. Palkovics L. Pogány M. (2018). Contribution of cell wall peroxidase- and NADPH oxidase -derived reactive oxygen species to *Alternaria brassicicola*-induced oxidative burst in *Arabidopsis*. *Mol. Plant Pathol.* 20 485–499. 10.1111/mpp.12769 30426643 PMC6637864

[B27] Kankam F. Long H. T. He J. Zhang C. H. Qiu H. (2016). 3-Methylthiopropionic Acid of *Rhizoctonia solani* AG-3 and its role in the pathogenicity of the fungus. *Plant Pathol. J.* 32 85–94. 10.5423/PPJ.OA.08.2015.0159 27147928 PMC4853098

[B28] Kaushik A. Roberts D. P. Ramaprasad A. Mfarrej S. Nair M. B. Lakshman D. (2020). The pangenome analysis of the soil-borne fungal phytopathogen *Rhizoctonia solani* and development of a comprehensive web resource: *Rsolani*DB. *biorxiv* [Preprint]. 10.1101/2020.12.18.423518 PMC8992008 35401459

[B29] King B. C. Waxman K. D. Nenni N. V. Walker L. P. Bergstrom G. C. Gibson D. M. (2011). Arsenal of plant cell wall degrading enzymes reflects host preference among plant pathogenic fungi. *Biotechnol. Biofuels* 4:4. 10.1186/1754-6834-4-4 21324176 PMC3051899

[B30] Kubicek C. P. Starr T. L. Glass N. L. (2014). Plant cell wall–degrading enzymes and their secretion in plant-pathogenic fungi. *Annu. Rev. Phytopathol.* 52 427–451. 10.1146/annurev-phyto-102313-045831 25001456

[B31] Kuninaga S. Carling D. E. Takeuchi T. Yokosawa R. (2000). Comparison of rDNA ITS sequences between potato and tobacco strains in *Rhizoctonia solani* AG-3. *J. Gen. Plant Pathol.* 66 2–11. 10.1007/PL00012917

[B32] Kuninaga S. Natsuaki T. Takeuchi T. Yokosawa R. (1997). Sequence variation of the rDNA ITS regions with between anastomosis groups in *Rhizoctonia solani*. *Curr. Genet.* 32 237–243. 10.1007/s002940050272 9339350

[B33] Lakshman D. K. Alkharouf N. Roberts D. P. Natarajan S. S. Mitra A. (2012). Gene expression profling of the plant pathogenic basidiomycetous fungus *Rhizoctonia solani* AG-4 reveals putative virulence factors. *Mycologia* 104 1020–1035. 10.3852/11-226 22778167

[B34] Langmead B. Salzberg S. (2012). Fast gapped-read alignment with Bowtie 2. *Nat. Methods* 9 357–359. 10.1038/nmeth.1923 22388286 PMC3322381

[B35] Li S. Peng X. Wang Y. Hua K. Y. Xing F. Zheng Y. Y. (2019). The effector *AGLIP1* in *Rhizoctonia solani* AG1 IA triggers cell death in plants and promotes disease development through inhibiting PAMP-triggered immunity in *Arabidopsis thaliana*. *Front. Microbiol*. 10:2228. 10.3389/fmicb.2019.02228 31611861 PMC6775501

[B36] Liboro K. Yu S.-R. Lim J. So Y.-S. Bahn Y.-S. Eoh H. (2021). Transcriptomic and metabolomic analysis revealed roles of Yck2 in Carbon metabolism and morphogenesis of *Candida albicans*. *Front. Cell. Infect. Microbiol.* 16:636834. 10.3389/fcimb.2021.636834 33796481 PMC8008151

[B37] Lightly T. J. Frejuk K. L. Groleau C. Chiarelli L. R. Cardona S. T. (2019). Phenylacetyl-CoA, not phenylacetic acid, attenuates CepIR-regulated virulence in *Burkholderia cenocepacia*. *Appl. Environ. Microbiol.* 85:e01594-19. 10.1128/AEM.01594-19 31585996 PMC6881814

[B38] Lightly T. J. Phung R. R. Sorensen J. L. Cardona S. T. (2017). Synthetic cystic fibrosis sputum medium diminishes *Burkholderia cenocepacia* antifungal activity against *Aspergillus fumigatus* independently of phenylacetic acid production. *Can. J. Microbiol.* 63 427–438. 10.1139/cjm-2016-0705 28178425

[B39] Liu M. Hu J. Zhang A. Dai Y. Chen W. He Y. (2021). Auxilin-like protein *MoSwa2* promotes effector secretion and virulence as a clathrin uncoating factor in the rice blast fungus *Magnaporthe oryzae*. *New Phytol.* 230 720–736. 10.1111/nph.17181 33423301 PMC8048681

[B40] Lo Presti L. Lanver D. Schweizer G. Tanaka S. Liang L. Tollot M. (2015). Fungal effectors and plant susceptibility. *Annu. Rev. Plant Biol.* 66 513–545. 10.1146/annurev-arplant-043014-114623 25923844

[B41] Lopez-Nieves S. Pringle A. Maeda H. A. (2019). Biochemical characterization of TyrA dehydrogenases from *Saccharomyces cerevisiae* (Ascomycota) and *Pleurotus ostreatus* (Basidiomycota). *Arch. Biochem. Biophys*. 665 12–19. 10.1016/j.abb.2019.02.005 30771296

[B42] Lucas G. B. (1975). *Diseases of tobacco*, 3rd Edn. Raleigh, NC: Biological Consulting Associates. 10.1038/1781421a0

[B43] McCarthy J. D. Chen Y. S. Smyth K. G. (2012). Differential expression analysis of multifactor RNA-Seq experiments with respect to biological variation. *Nucleic Acids Res.* 40 4288–4297. 10.1093/nar/gks042 22287627 PMC3378882

[B44] Mohammad-Saeid J. Juan-Francisco M. Carlos B. Rebeca D. S. María-Fernanda V. C. María P. (2018). Catabolism of phenylacetic acid in *Penicillium rubens*. Proteome-wide analysis in response to the benzylpenicillin side chain precursor. *J. Proteomics* 187 243–259. 10.1016/j.jprot.2018.08.006 30092379

[B45] Moore K. Rao P. V. Towers G. H. N. (1968). Degradation of Phenylalanine and Tyrosine by *Sporobolomyces roseus*. *Biochem. J.* 106 507–514. 10.1042/bj1060507 5688927 PMC1198531

[B46] Moore K. Towers G. H. N. (1967). Degradation of Aromatic Amino Acids By Fungi I. Fate of L-Phenylalanine in *Schizophyllum* commune. *Can. J. Biochem.* 45 1659–1665. 10.1139/o67-196 6070754

[B47] Ogoshi A. (1987). Ecology and pathogenicity of anastomosis and intraspecifc groups of *Rhizoctonia Solani* Kühn. *Annu. Rev. Phytopathol.* 25 125–143. 10.1146/annurev.py.25.090187.001013

[B48] Pauly P. N. (2012). A burst of plant NADPH oxidases. *Trends Plant Sci.* 17 9–15. 10.1016/j.tplants.2011.10.001 22037416

[B49] Qin T. F. Hao W. Sun R. R. Li Y. Q. (2020). *Verticillium dahliae* VdTHI20, involved in pyrimidine biosynthesis, is required for DNA repair functions and pathogenicity. *Int. J. Mol. Sci.* 20:1378. 10.3390/ijms21041378 32085660 PMC7073022

[B50] Robinson M. D. McCarthy D. J. Smyth G. K. (2010). edgeR: A Bioconductor package for differential expression analysis of digital gene expression data. *Bioinformatics* 26 139–140. 10.1093/bioinformatics/btp616 19910308 PMC2796818

[B51] Shew H. D. Main C. E. (1985). *Rhizoctonia* leaf spot of flue-cured tobacco in North Carolina. *Plant Dis.* 69 901–903.

[B52] Siddiqui I. A. Shaukat S. S. (2005). Phenylacetic acid-producing *Rhizoctonia solani* represses the biosynthesis of nematicidal compounds in vitro and influences biocontrol of meloidogyne incognita in tomato by *Pseudomonas fluorescens* Strain CHA0 and its GM Derivatives. *J. Appl. Microbiol.* 98 43–55. 10.1111/j.1365-2672.2004.02457.x 15610416

[B53] Slot J. C. Rokas A. (2010). Multiple GAL pathway gene clusters evolved independently and by different mechanisms in fungi. *Proc. Natl. Acad. Sci. U.S.A.* 107 10136–10141. 10.1073/pnas.0914418107 20479238 PMC2890473

[B54] Sneh B. Jajabi-Hare S. Neate S. Dijst G. (1996). *Rhizoctonia species: Taxonomy, molecular biology, ecology, pathology and disease control.* Dordrecht: Kluwer Academic Publishers.

[B55] Sperschneider J. Dodds P. N. Gardiner D. M. Singh K. B. Taylor J. M. (2018). Improved prediction of fungal efector proteins from secretomes with EfectorP 2.0. *Mol. Plant Pathol.* 19 2094–2110. 10.1111/mpp.12682 29569316 PMC6638006

[B56] Stergiopoulos I. de Wit P. J. (2009). Fungal effector proteins. *Annu. Rev. Phytopathol.* 47 233–263. 10.1146/annurev.phyto.112408.132637 19400631

[B57] Taheri P. Tarighi S. (2011). Cytomolecular aspects of rice sheath blight caused by *Rhizoctonia solani*. *Eur. J. Plant Pathol.* 129 511–528. 10.1007/s10658-010-9725-7

[B58] Vidhyasekaran P. Ponmalar T. R. Samiyappan R. Velazhahan R. Muthukrishnan S. (1997). Host-specifc toxin production by *Rhizoctonia solani*, the Rice Sheath Blight Pathogen. *Phytopathology* 87 1258–1263. 10.1094/PHYTO.1997.87.12.1258 18945027

[B59] Wang C. Pi L. Jiang S. Yang M. Shu C. Zhou E. (2018). ROS and trehalose regulate sclerotial development in *Rhizoctonia solani* AG-1 IA. *Fungal Biol.* 122 322–332. 10.1016/j.funbio.2018.02.003 29665958

[B60] Wu J. Mao X. Cai T. Luo J. Wei L. (2006). KOBAS server: A web-based platform for automated annotation and pathway identification. *Nucleic Acids Res.* 34 720–724. 10.1093/nar/gkl167 16845106 PMC1538915

[B61] Wu Y. H. Zhao Y. Q. Fu Y. Zhao X. X. Chen J. G. (2012). First report of target spot of flue-cured Tobacco Caused by *Rhizoctonia solani* AG-3 in China. *Plant Dis.* 96 1824–1824. 10.1094/PDIS-06-12-0551-PDN 30727290

[B62] Xia Y. Ma Z. Qiu M. Guo B. Wang Y. (2020). N-glycosylation shields *Phytophthora sojae* apoplastic effector *PsXEG1* from a specific host aspartic protease. *Proc. Natl. Acad. Sci. U.S.A.* 117 27685–27693. 10.1073/pnas.2012149117 33082226 PMC7959567

[B63] Xu C. T. Zhang C. Zhang M. J. (2021). The study on pathogen identification and biological control of tobacco target spot in Sichuan Province. *Hubei Agric. Sci. China* 60:4. 10.14088/j.cnki.issn0439-8114.2021.08.016

[B64] Xu M. L. Hao K. Q. Yang J. G. Wang F. L. Xiao Z. X. Li W. (2018). First Report of *Rhizoctonia solani* AG-3 Causing Tobacco Target Spot in Yunnan, China. *Plant Dis*. 102:2038. 10.1094/PDIS-02-18-0249-PDN 30113255

[B65] Yamamoto N. Wang Y. Lin R. Liang Y. Liu Y. Zhu J. (2019). Integrative transcriptome analysis discloses the molecular basis of a heterogeneous fungal phytopathogen complex, *Rhizoctonia solani* AG-1 subgroups. *Sci. Rep.* 9:19626. 10.1038/S41598-019-55734-2 31873088 PMC6928066

[B66] Yang G. H. Conner R. L. Chen Y. Y. Chen J. Y. Wang Y. G. (2008). Frequency and pathogenicity distribution of *Rhizoctona* spp. causing sheath blight on rice and banded leaf disease on Maize in Yunnan, China. *J. Plant Pathol.* 90 387–392. 10.4454/jpp.v90i2.679 32896216

[B67] Zhang J. Chen L. Fu C. Wang L. Liu H. Cheng Y. (2017). Comparative transcriptome analyses of gene expression changes triggered by *Rhizoctonia solani* AG1 IA infection in resistant and susceptible rice varieties. *Front. Plant Sci.* 8:1422. 10.3389/fpls.2017.01422 28861102 PMC5562724

[B68] Zhang M. Rajput N. A. Shen D. Peng S. Zeng W. Liu T. (2015). A Phytophthora sojae cytoplasmic effector mediates disease resistance and abiotic stress tolerance in *Nicotiana benthamiana*. *Sci. Rep.* 5:10837. 10.1038/srep10837 26039925 PMC4454142

[B69] Zheng A. Lin R. Zhang D. Qin P. Xu L. Ai P. (2013). The evolution and pathogenic mechanisms of the rice sheath blight pathogen. *Nat. Commun.* 4:1424. 10.1038/ncomms2427 23361014 PMC3562461

