# Supplementary material for: Integrative transcriptome analysis revealed the pathogenic molecular basis of Rhizoctonia solani AG-3 TB at three progressive stages of infection
Source: Front Microbiol. 2022 Oct 11;13:1001327. doi: 10.3389/fmicb.2022.1001327 (PMC9593035; doi:10.3389/fmicb.2022.1001327)
Supplement: Supplementary file 1 [file Data_Sheet_1.docx]

**Supplementary Materials**

**Table S1.** Sequence primer of qPCR gene.

| Gene name | Forward primer | Reverse primer |
| --- | --- | --- |
| TRINITY_DN14681_c0_g1_i2 | GTTTGGCGTTCAAGTCACCC | GCGTCACTCTCGATGGCATA |
| TRINITY_DN17797_c0_g2_i1 | CGACAACGCTTGGCTTTGAA | GTACCTCCACCAGTACCCCT |
| TRINITY_DN29708_c0_g1_i1 | CCACCGAGAAGCCTACCATC | TGCAGCAAGAGAGGTGACAG |
| TRINITY_DN3377_c0_g1_i1 | AATTGCCACTCTCGCTTCTG | ACCGTTGACGAAGAAAGTGC |
| TRINITY_DN20778_c0_g1_i1 | TTCCATCAACACGGTCCTCG | TCAGACTCTTGTCCGGCAAC |
| TRINITY_DN18673_c0_g3_i1 | CGCATTTCGCTACGAACCAG | TCCTTGGGTCAATGAGGTGC |
| TRINITY_DN21085_c0_g1_i1 | CGTTCACAAGGAAGTCCGGA | CTGAACGCGGGCATTTTGAA |
| TRINITY_DN20404_c0_g2_i1 | CTGTGATGGGTGTCAACGGA | GGTAAAAGTGGTCCCGCTCA |
| TRINITY_DN21213_c0_g1_i1 | CCTGGGACGTCGTCAATGAA | TTCACCGCAGCATCAGTCTT |
| TRINITY_DN18070_c0_g1_i4 | AATTCACGACTCGTTGCCCT | TTGTCAAAGACGGGCTCTCC |
| Actin | CCAACCGAGAAAAGATGACGC | CGTAAATTGGAACCGTATGCG |

**Table S2.** The number of DEGs at different infection stages of *R. solani* AG-3 TB.

| stage of infection | *R. solani* AG-3 TB | | |
| --- | --- | --- | --- |
|  | up | down | total |
|  |  |  |  |
| 6 hpi | 9471 | 7836 | 17307 |
| 12 hpi | 8846 | 11846 | 20692 |
| 24 hpi | 13876 | 7192 | 21068 |
| 36 hpi | 14257 | 8046 | 22303 |
| 48 hpi | 11153 | 4517 | 15670 |
| 72 hpi | 15025 | 6770 | 21795 |

**Table S3.** Screening of candidate pathogenic effector proteins, including 13 cytoplasmic effectors and 28 apoplastic effectors.

| Effector | Geneid | Transid | Pfam | Blast | Reads |
| --- | --- | --- | --- | --- | --- |
| Cytoplasmic effectors | TRINITY_DN7546_c0_g1 | TRINITY_DN7546_c0_g1_i1 | Domain of unknown function (DUF1771) | Smr domain protein | 103 |
|  | TRINITY_DN17844_c0_g3 | TRINITY_DN17844_c0_g3_i1 | Peptidase inhibitor I9 | serine protease | 229 |
|  | TRINITY_DN18186_c0_g4 | TRINITY_DN18186_c0_g4_i1 | OHCU decarboxylase | vitamin-D-receptor interacting mediator subunit 4 | 136 |
|  | TRINITY_DN18333_c0_g1 | TRINITY_DN18333_c0_g1_i5 | Putative quorum-sensing-regulated virulence factor | putative Ran-binding protein | 267 |
|  | TRINITY_DN19259_c0_g1 | TRINITY_DN19259_c0_g1_i2 | EF-hand domain | unnamed protein product | 1150 |
|  | TRINITY_DN19398_c0_g2 | TRINITY_DN19398_c0_g2_i2 | RNA recognition motif. (a.k.a. RRM, RBD, or RNP domain) | RNA recognition motif 1 in RNA-binding protein | 533 |
|  | TRINITY_DN20404_c0_g2 | TRINITY_DN20404_c0_g2_i1 | Egh16-like virulence factor | DUF3129 family protein | 499 |
|  | TRINITY_DN20859_c0_g1 | TRINITY_DN20859_c0_g1_i1 | Ubiquinol-cytochrome-c reductase complex subunit (QCR10) | hypothetical protein RSOL_496070 | 149 |
|  | TRINITY_DN22450_c1_g5 | TRINITY_DN22450_c1_g5_i2 | Eukaryotic metallothionein | hypothetical protein V565_107130 | 293 |
|  | TRINITY_DN21987_c0_g1 | TRINITY_DN21987_c0_g1_i4 | Type II site-specific deoxyribonuclease | hypothetical protein V565_135530 | 1030 |
|  | TRINITY_DN22565_c0_g4 | TRINITY_DN22565_c0_g4_i1 | Methyl-CpG binding domain | unnamed protein product | 534 |
|  | TRINITY_DN20094_c1_g2 | TRINITY_DN20094_c1_g2_i1 | 60s Acidic ribosomal protein | unnamed protein product | 36001 |
|  | TRINITY_DN19541_c0_g1 | TRINITY_DN19541_c0_g1_i1 | Ribosomal protein S6 | haloacid dehalogenase-like hydrolase | 113 |
| Apoplastic effectors | TRINITY_DN2386_c0_g1 | TRINITY_DN2386_c0_g1_i1 | Putative ephrin-receptor like | GCC2 and GCC3 domain protein | 105 |
|  | TRINITY_DN2928_c0_g1 | TRINITY_DN2928_c0_g1_i1 | Phosphatidylethanolamine-binding protein | OV-16 antigen | 283 |
|  | TRINITY_DN3377_c0_g1 | TRINITY_DN3377_c0_g1_i1 | Polysaccharide deacetylase | carbohydrate esterase family 4 protein | 1989 |
|  | TRINITY_DN6754_c0_g1 | TRINITY_DN6754_c0_g1_i1 | - | hypothetical protein BN14_10972 | 97 |
|  | TRINITY_DN7106_c0_g1 | TRINITY_DN7106_c0_g1_i1 | Esterase PHB depolymerase | acetylxylan esterase, putative | 596 |
|  | TRINITY_DN10220_c0_g1 | TRINITY_DN10220_c0_g1_i1 | Pectinesterase | pectinesterase | 404 |
|  | TRINITY_DN12825_c0_g1 | TRINITY_DN12825_c0_g1_i2 | Glycosyl hydrolases family 28 | endo-polygalacturonase PG1 | 352 |
|  | TRINITY_DN13024_c0_g3 | TRINITY_DN13024_c0_g3_i2 | Protein of unknown function (DUF3455) | putative malate dehydrogenase | 234 |
|  | TRINITY_DN15705_c0_g1 | TRINITY_DN15705_c0_g1_i1 | Glycosyl hydrolase family 61 | glycoside hydrolase family 61 protein | 92 |
|  | TRINITY_DN17042_c0_g2 | TRINITY_DN17042_c0_g2_i1 | Pentapeptide repeats (8 copies) | unnamed protein product | 129 |
|  | TRINITY_DN17535_c0_g1 | TRINITY_DN17535_c0_g1_i1 | Glycosyl hydrolases family 28 | glycoside hydrolase family 28 protein | 152 |
|  | TRINITY_DN17851_c0_g1 | TRINITY_DN17851_c0_g1_i1 | Pectinesterase | pectin methylesterase family protein, partial | 101 |
|  | TRINITY_DN18333_c0_g1 | TRINITY_DN18333_c0_g1_i1 | Putative quorum-sensing-regulated virulence factor | putative Ran-binding protein | 267 |
|  | TRINITY_DN18673_c0_g2 | TRINITY_DN18673_c0_g2_i1 | Pregnancy-associated plasma protein-A | extracellular metalloprotease | 248 |
|  | TRINITY_DN18673_c0_g3 | TRINITY_DN18673_c0_g3_i1 | Pregnancy-associated plasma protein-A | extracellular metalloprotease | 2730 |
|  | TRINITY_DN19525_c0_g1 | TRINITY_DN19525_c0_g1_i1 | Lysine-specific metallo-endopeptidase | deuterolysin metalloprotease (M35) family containing protein | 1433 |
|  | TRINITY_DN20262_c0_g1 | TRINITY_DN20262_c0_g1_i1 | Subtilase family | S8/S53 family peptidase | 77 |
|  | TRINITY_DN20721_c0_g1 | TRINITY_DN20721_c0_g1_i1 | WSC domain | glycoside hydrolase family 16 protein | 146 |
|  | TRINITY_DN21592_c0_g1 | TRINITY_DN21592_c0_g1_i1 | Surfeit locus protein 6 | glycosyl hydrolase family 43 protein | 130 |
|  | TRINITY_DN21690_c1_g9 | TRINITY_DN21690_c1_g9_i1 | Glycosyl hydrolase family 7 | putative 1,4-beta-D-glucan cellobiohydrolase B | 122 |
|  | TRINITY_DN22002_c0_g4 | TRINITY_DN22002_c0_g4_i1 | Chitin binding Peritrophin-A domain | pria protein | 156 |
|  | TRINITY_DN22904_c0_g1 | TRINITY_DN22904_c0_g1_i1 | - | pectin lyase F, putative | 163 |
|  | TRINITY_DN7175_c0_g1 | TRINITY_DN7175_c0_g1_i1 | Protease inhibitor/seed storage/LTP family | non-specific lipid-transfer protein 1-like | 100 |
|  | TRINITY_DN18021_c0_g1 | TRINITY_DN18021_c0_g1_i3 | Lytic transglycolase | rare lipoprotein A-like double-psi beta-barrel protein, partial | 579 |
|  | TRINITY_DN20527_c0_g1 | TRINITY_DN20527_c0_g1_i2 | Sar8.2 family | uncharacterized protein LOC104086212 | 1342 |
|  | TRINITY_DN21085_c0_g1 | TRINITY_DN21085_c0_g1_i1 | Potato type II proteinase inhibitor family | proteinase inhibitor type-2 | 2063 |
|  | TRINITY_DN8852_c0_g1 | TRINITY_DN8852_c0_g1_i1 | Transglycosylase SLT domain | glycoside hydrolase family 23 protein | 4795 |
|  | TRINITY_DN18103_c0_g2 | TRINITY_DN18103_c0_g2_i1 | - | hypothetical protein RSOLAG22IIIB_02861 | 88 |

**Table S4.** Small cysteine-rich proteins secretion in *R. solani* AG-3 TB infection

| Geneid | Amino acid size | Cysteine ratio(％) | Blast |
| --- | --- | --- | --- |
| TRINITY_DN939_c0_g1 | 87 | 9.2 | hypothetical protein AG1IA_05894 |
| TRINITY_DN2347_c0_g1 | 82 | 9.8 | - |
| TRINITY_DN2411_c0_g1 | 46 | 10.9 | - |
| TRINITY_DN2776_c0_g1 | 78 | 6.4 | - |
| TRINITY_DN4361_c0_g1 | 94 | 6.4 | 14 kDa proline-rich protein DC2.15-like |
| TRINITY_DN6348_c0_g1 | 83 | 6.0 | - |
| TRINITY_DN7144_c0_g2 | 144 | 4.2 | DNA-damage-repair/toleration protein DRT100-like |
| TRINITY_DN7175_c0_g1 | 118 | 7.6 | non-specific lipid-transfer protein 1-like |
| TRINITY_DN7725_c0_g1 | 162 | 7.4 | unnamed protein product |
| TRINITY_DN7952_c0_g1 | 122 | 4.1 | Homo sapiens chemokine (C-C motif) ligand 2, partial |
| TRINITY_DN8540_c0_g1 | 48 | 14.6 | - |
| TRINITY_DN9207_c0_g1 | 83 | 6.0 | chloroperoxidase |
| TRINITY_DN9235_c0_g1 | 254 | 6.3 | unnamed protein product |
| TRINITY_DN10229_c0_g1 | 97 | 5.2 | uncharacterized protein |
| TRINITY_DN10765_c0_g1 | 193 | 13.0 | unnamed protein product |
| TRINITY_DN11789_c0_g1 | 153 | 4.6 | unnamed protein product |
| TRINITY_DN12236_c0_g1 | 115 | 5.2 | butyrophilin-like protein 1-like |
| TRINITY_DN12691_c0_g3 | 97 | 6.2 | unnamed protein product |
| TRINITY_DN14020_c0_g1 | 184 | 6.0 | unnamed protein product |
| TRINITY_DN14593_c0_g1 | 39 | 15.4 | - |
| TRINITY_DN16017_c0_g1 | 41 | 4.9 | - |
| TRINITY_DN16826_c0_g1 | 226 | 7.1 | pathogenesis-related protein R major form precursor |
| TRINITY_DN17236_c0_g1 | 133 | 5.3 | unnamed protein product |
| TRINITY_DN17871_c0_g3 | 118 | 6.8 | glycoside hydrolase family 18 protein, partial |
| TRINITY_DN18021_c0_g1 | 213 | 4.7 | rare lipoprotein A-like double-psi beta-barrel protein, partial |
| TRINITY_DN18103_c0_g2 | 182 | 4.4 | unnamed protein product |
| TRINITY_DN18199_c0_g1 | 150 | 4.0 | pathogenesis-related protein PR5K |
| TRINITY_DN18417_c0_g1 | 65 | 7.7 | unnamed protein product |
| TRINITY_DN19817_c0_g2 | 246 | 7.3 | unnamed protein product |
| TRINITY_DN20527_c0_g1 | 123 | 10.6 | uncharacterized protein |
| TRINITY_DN21085_c0_g1 | 197 | 12.2 | proteinase inhibitor type-2 |
| TRINITY_DN22117_c0_g3 | 48 | 6.2 | - |
| TRINITY_DN22660_c4_g8 | 78 | 9.0 | hypothetical protein DJ568_17050, partial |
| TRINITY_DN24062_c0_g1 | 44 | 9.1 | - |
| TRINITY_DN24725_c0_g1 | 72 | 8.3 | unnamed protein product |
| TRINITY_DN25484_c0_g1 | 92 | 9.8 | - |
| TRINITY_DN25934_c0_g1 | 81 | 6.2 | - |
| TRINITY_DN27004_c0_g1 | 59 | 5.1 | - |
| TRINITY_DN27087_c0_g1 | 111 | 5.4 | probably inactive leucine-rich repeat receptor-like protein kinase At5g48380 |
| TRINITY_DN27958_c0_g1 | 97 | 6.2 | hypothetical protein SASPL_145633 |
| TRINITY_DN31515_c0_g1 | 41 | 4.9 | zinc finger protein |
| TRINITY_DN32756_c0_g1 | 43 | 4.7 | - |
| TRINITY_DN33187_c0_g1 | 74 | 5.4 | plasma membrane fusion-related protein |
| TRINITY_DN34591_c0_g1 | 63 | 12.7 | - |
| TRINITY_DN34787_c0_g1 | 93 | 9.7 | MYND finger protein |
| TRINITY_DN35894_c0_g1 | 97 | 7.2 | arp2/3 complex-activating protein rickA-like |
| TRINITY_DN3697_c0_g1 | 40 | 5.0 | - |
| TRINITY_DN6025_c0_g1 | 93 | 4.3 | neurotrypsin, partial |
| TRINITY_DN6474_c0_g1 | 127 | 5.5 | glycoside hydrolase family 5 protein |
| TRINITY_DN8023_c0_g1 | 103 | 9.7 | - |
| TRINITY_DN8047_c0_g1 | 81 | 2.5 | serine incorporator 1 isoform X2 |
| TRINITY_DN8378_c0_g1 | 70 | 4.3 | hCG2044994 |
| TRINITY_DN10745_c0_g1 | 93 | 4.3 | - |
| TRINITY_DN13691_c0_g1 | 117 | 8.5 | unnamed protein product |
| TRINITY_DN15103_c0_g1 | 87 | 7.0 | PREDICTED: chitinase-like protein 1 |
| TRINITY_DN15147_c0_g2 | 80 | 5.0 | - |
| TRINITY_DN15573_c0_g1 | 92 | 7.6 | non-specific lipid transfer protein GPI-anchored 1 |
| TRINITY_DN15832_c0_g1 | 120 | 5.0 | unnamed protein product |
| TRINITY_DN19541_c0_g1 | 162 | 6.2 | unnamed protein product |
| TRINITY_DN20021_c0_g2 | 67 | 6.0 | - |
| TRINITY_DN20094_c1_g2 | 113 | 8.8 | unnamed protein product |
| TRINITY_DN21025_c0_g2 | 131 | 6.9 | - |
| TRINITY_DN23018_c0_g1 | 163 | 4.3 | MET isoform 7, partial |
| TRINITY_DN23209_c0_g1 | 103 | 7.8 | - |
| TRINITY_DN24304_c0_g1 | 76 | 5.3 | - |
| TRINITY_DN26140_c0_g1 | 114 | 6.2 | - |
| TRINITY_DN27210_c0_g1 | 90 | 5.6 | - |
| TRINITY_DN28740_c0_g1 | 98 | 6.1 | fungal specific transcription factor domain protein |
| TRINITY_DN29395_c0_g1 | 68 | 7.4 | unnamed protein product |
| TRINITY_DN29887_c0_g1 | 133 | 4.5 | rapid alkalinization factor-like |
| TRINITY_DN29932_c0_g1 | 86 | 4.7 | hypothetical protein FRC11_008866 |
| TRINITY_DN30715_c0_g1 | 114 | 7.9 | plasminogen activator, urokinase receptor, isoform CRA_a, partial |
| TRINITY_DN30849_c0_g1 | 47 | 6.4 | - |
| TRINITY_DN31731_c0_g1 | 106 | 7.5 | prosaposin-like |
| TRINITY_DN33697_c0_g1 | 49 | 6.1 | - |
| TRINITY_DN34440_c0_g1 | 162 | 4.3 | KIAA0368 protein |
| TRINITY_DN34787_c0_g1 | 93 | 9.7 | MYND finger protein |
| TRINITY_DN35894_c0_g1 | 97 | 7.2 | arp2/3 complex-activating protein rickA-like |

Note: the “-” represents no blast results.

**Table S5**. The mapped rates of the sequencing data with the reference genomes AG3-T5.

| Sample | Total reads | Total mapped (%) | Non-unique (%) | Unique (%) | Unmapped reads (%) |
| --- | --- | --- | --- | --- | --- |
| CK_R_48h_1 | 39064234 | 15674215(40.12) | 933333(5.95) | 14740882(94.05) | 23390019(59.88) |
| CK_R_48h_2 | 36796412 | 14871757(40.42) | 930588(6.26) | 13941169(93.74) | 21924655(59.58) |
| CK_R_48h_3 | 41597428 | 16740659(40.24) | 1009970(6.03) | 15730689(93.97) | 24856769(59.76) |


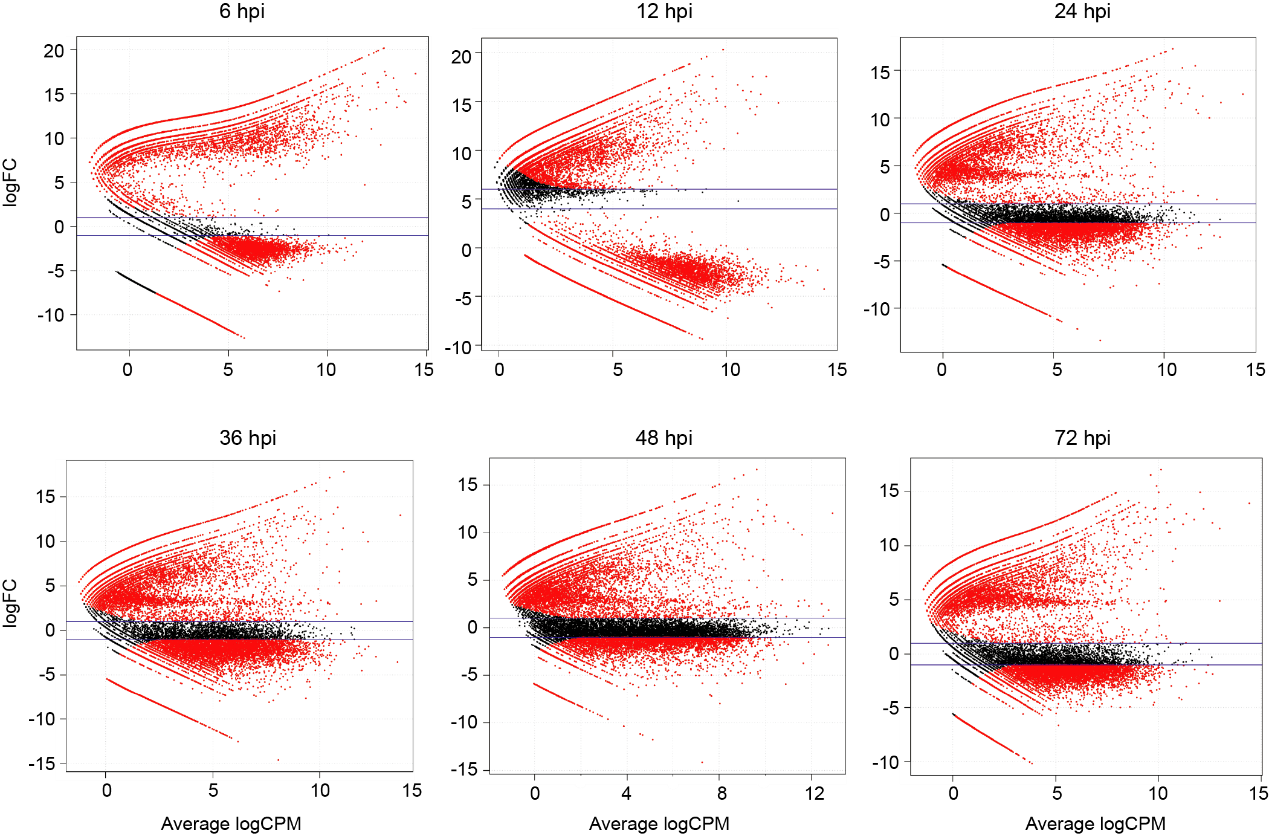


**Figure S1.** MA-plots generated for the DEGs of different infection stage for *R. solani* AG-3 TB. The genes (logFC-scale) for candidate DEGs represented in MA-plots (numerals indicate hours after inoculation onto tobacco).


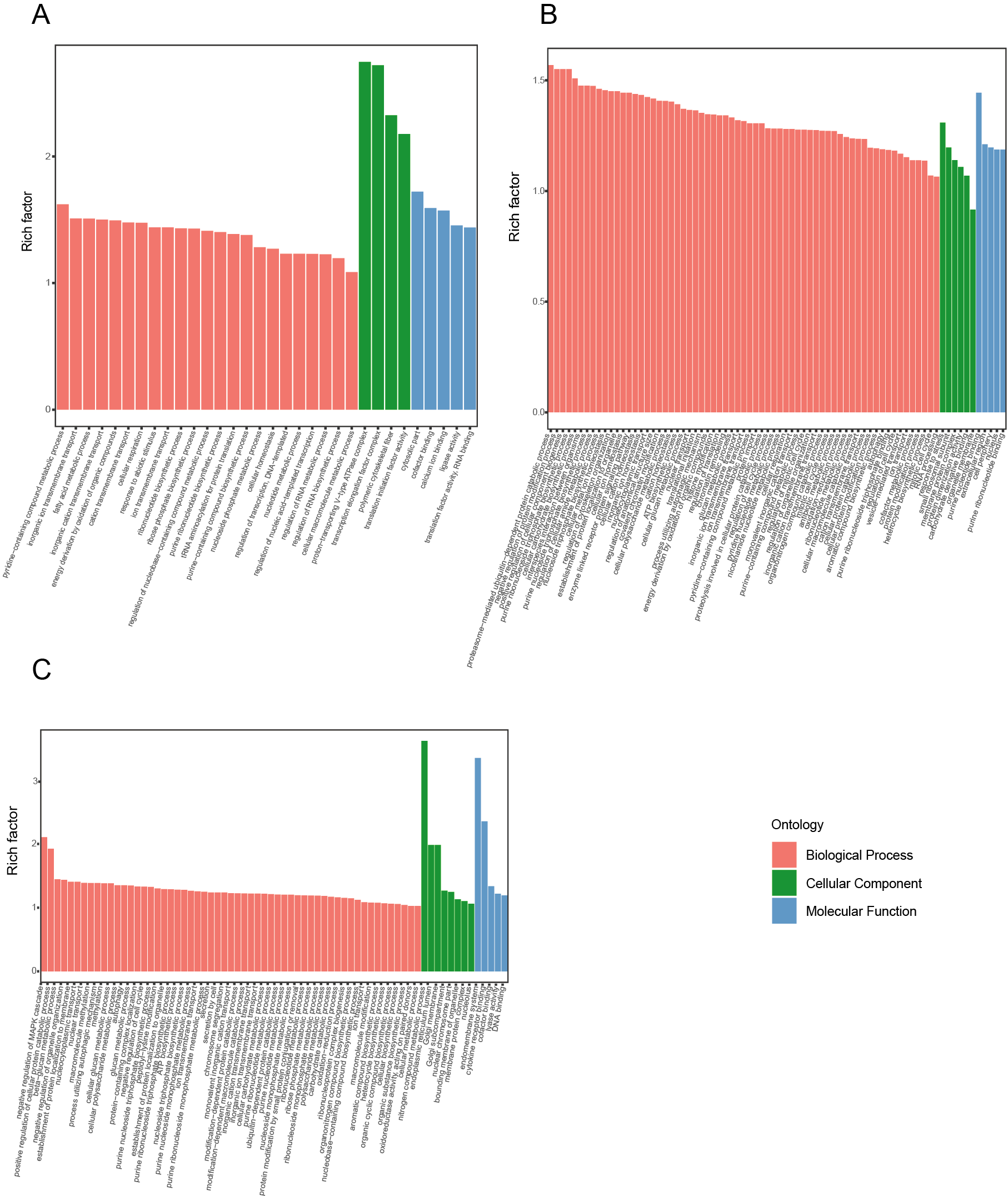


**Figure S2.** GO items pathway analysis the DEGs of *R. solani* AG-3 TB at different infection stages. (A) GO items enrichment analysis at 6 hpi stage. (B) GO items enrichment analysis at 24 hpi stage. (C) GO items enrichment analysis at 72 hpi stage.


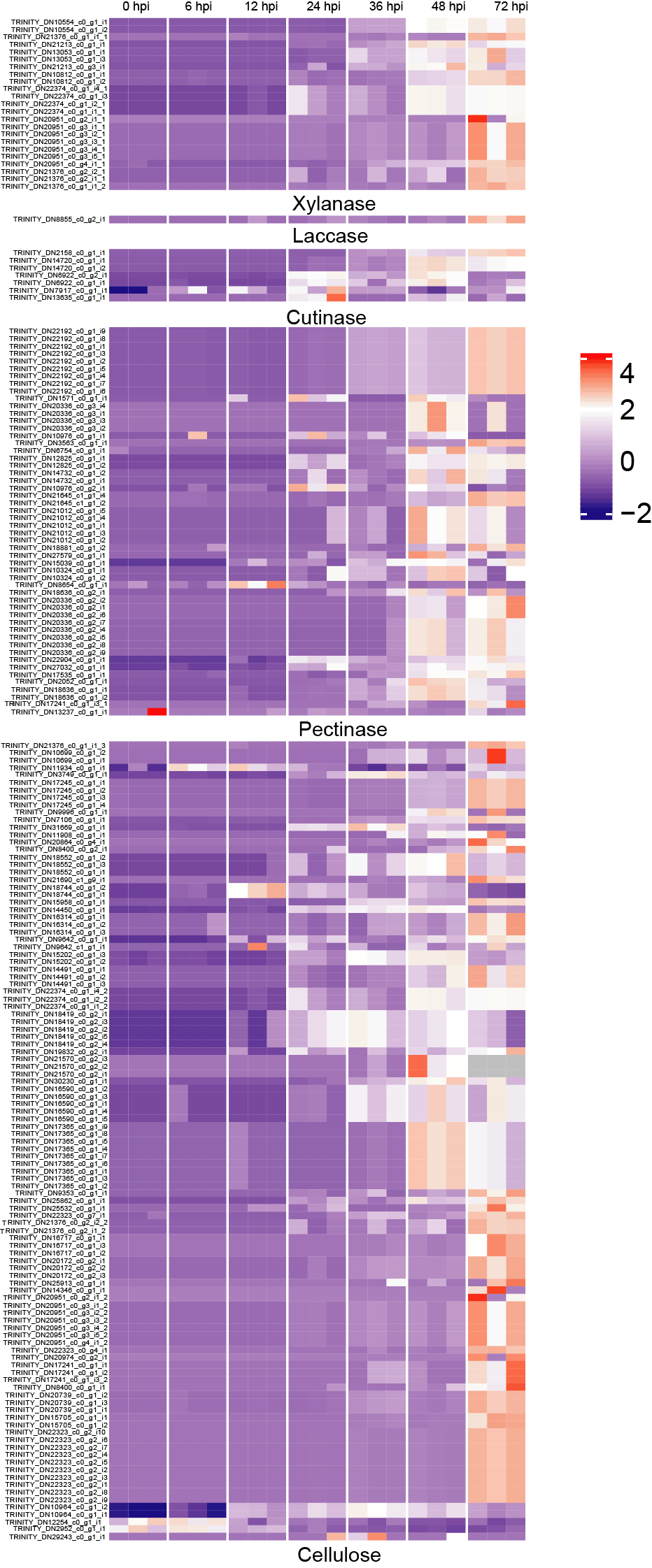


**Figure S3.** The heatmap of the expression in CWDEs genes in different stages. Expression patterns of candidate genes (RPKM in log10-scale) for CWDEs represented in heatmaps (numerals indicate hours after inoculation onto tobacco).


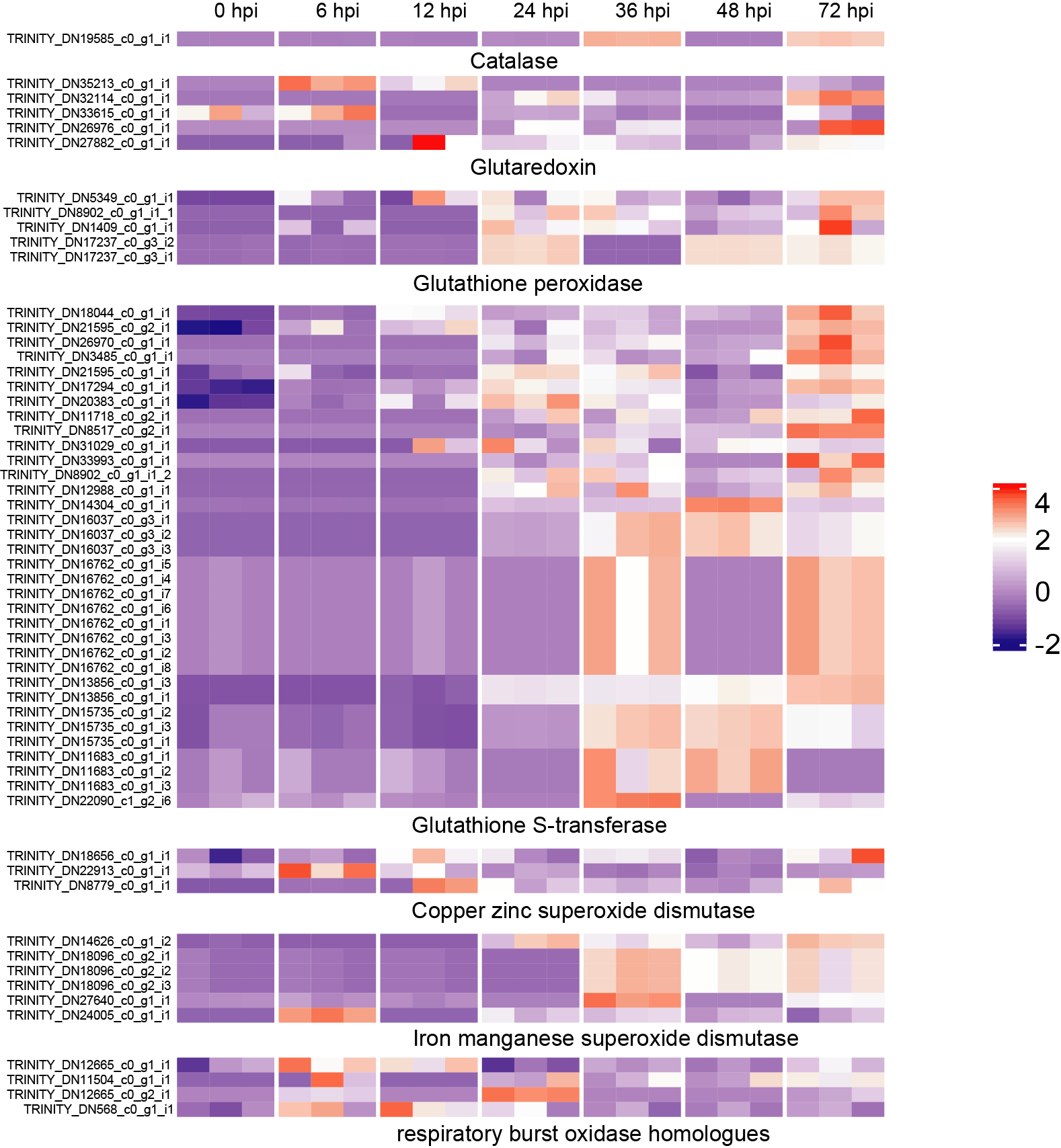


**Figure S4.** The heatmap showed the expression patterns of respiratory burst oxidase homologues in different stages (RPKM in log10-scale).


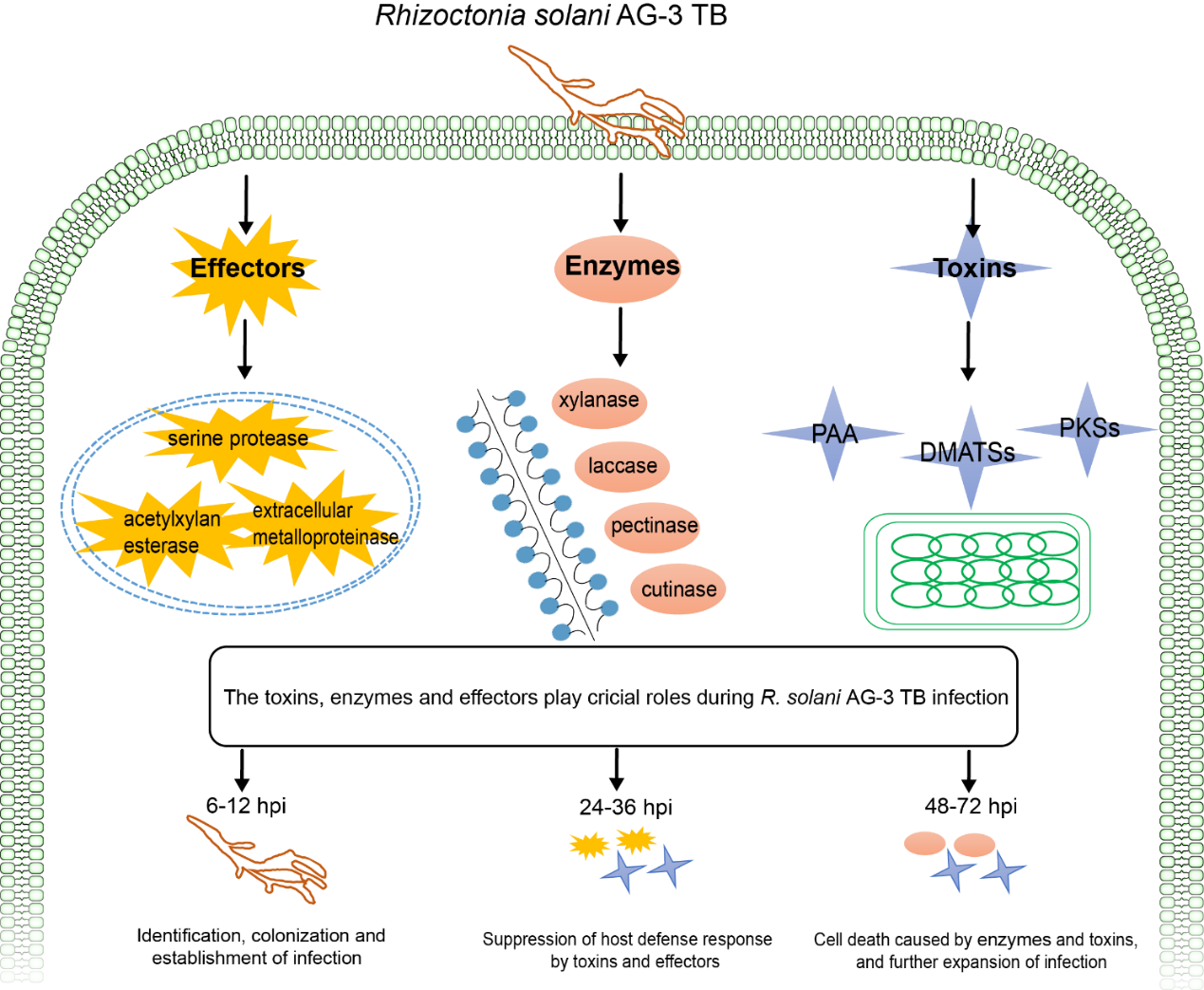


**Figure S5.** A hypothetical model for critical roles during *R. solani* AG-3 TB infection. The critical roles including toxins, enzymes and effectors play the different function at three infection stages (6-12 hpi, 24-36 hpi and 48-72 hpi).
